# The Factor Structure of Cognitive Functioning in Cognitively Healthy Participants: a Meta-Analysis and Meta-Analysis of Individual Participant Data

**DOI:** 10.1007/s11065-019-09423-6

**Published:** 2020-02-01

**Authors:** Joost A. Agelink van Rentergem, Nathalie R. de Vent, Ben A. Schmand, Jaap M. J. Murre, Janneke P. C. Staaks, ANDI Consortium, Hilde M. Huizenga

**Affiliations:** 1 Department of Psychology, University of Amsterdam, Nieuwe Achtergracht 129B, 1018WS Amsterdam, The Netherlands; 2 Department of Psychosocial Research and Epidemiology, Netherlands Cancer Institute, Amsterdam, The Netherlands; 3 Department of Medical Psychology, Academic Medical Center, Amsterdam, The Netherlands; 4 Amsterdam Brain and Cognition Center Amsterdam, University of Amsterdam, Amsterdam, The Netherlands; 5 Research Institute of Child Development and Education, University of Amsterdam, Amsterdam, The Netherlands; 6 Research priority area Yield, University of Amsterdam, Amsterdam, The Netherlands

**Keywords:** Factor analysis, Meta-analysis of individual participant data, Meta-analytic SEM, Clinical neuropsychology, Cognitive functioning, Neuropsychological tests, Cattell-horn-Carroll model

## Abstract

Many neuropsychologists are of the opinion that the multitude of cognitive tests may be grouped into a much smaller number of cognitive domains. However, there is little consensus on how many domains exist, what these domains are, nor on which cognitive tests belong to which domain. This incertitude can be solved by factor analysis, provided that the analysis includes a broad range of cognitive tests that have been administered to a very large number of people. In this article, two such factor analyses were performed, each combining multiple studies. However, because it was not possible to obtain complete multivariate data on more than the most common test variables in the field, not all possible domains were examined here. The first analysis was a factor meta-analysis of correlation matrices combining data of 60,398 healthy participants from 52 studies. Several models from the literature were fitted, of which a version based on the Cattell-Horn-Carroll (CHC) model was found to describe the correlations better than the others. The second analysis was a factor analysis of the Advanced Neuropsychological Diagnostics Infrastructure (ANDI) database, combining scores of 11,881 participants from 54 Dutch and Belgian studies not included in the first meta-analysis. Again, the model fit was better for the CHC model than for other models. Therefore, we conclude that the CHC model best characterizes both cognitive domains and which test belongs to each domain. Therefore, although originally developed in the intelligence literature, the CHC model deserves more attention in neuropsychology.

**Electronic supplementary material:**

The online version of this article (10.1007/s11065-019-09423-6) contains supplementary material, which is available to authorized users.

Neuropsychological tests are designed to measure cognitive functions, which may be impaired by brain disorders like Alzheimer’s or Parkinson’s disease, traumatic brain injury, or stroke. The tests neuropsychologists use are often assigned to various cognitive domains such as executive function, memory, or attention.

There are many reasons for establishing domains of cognitive functions and for assigning tests to these domains. The first reason may be that a clinician suspects problems in a specific cognitive domain for a particular patient and wants to select tests from this domain to administer. For example, if a patient comes in with subjective memory complaints, memory could be investigated further by selecting tests from this domain. The second reason may be that a clinician wants to qualify whether a particular patient is suffering from impairment on a single domain or on multiple domains. In the literature on mild cognitive impairment (MCI), for example, single-domain or multi-domain MCI are considered separate diagnoses, which have separate prognoses (Petersen, 2004). The third reason may be that a clinician or researcher wants to use composite scores on cognitive domains as an outcome measure rather than separate test scores. This method can reduce noise from individual measurement instruments (but see Lezak, Howieson, Bigler, & Tranel, 2012, p. 159). These composite scores may be calculated by summing the scores of individual tests that belong to a particular domain, as is done in the calculation of Perceptual Reasoning or Verbal Comprehension. A more sophisticated approach is to obtain estimates of a latent variable through factor analysis or item response theory analysis of a single domain and use scores on the latent variable as an outcome measure (Gross et al., 2015). The fourth reason may be to establish the validity of a particular test. If a researcher designs a new test intended to measure memory, he or she can calculate whether scores correlate highly with other tests in the memory domain, and do not correlate as highly with tests from other domains. Therefore, domains can be used to show both convergent and divergent validity. The fifth reason may be to handle missing value problems, such as those encountered in the Advanced Neuropsychological Diagnostics Infrastructure (ANDI) project (De Vent et al., 2016a). In this project, neuropsychological healthy control group data of many studies are combined to provide a normative database to be used in normative comparisons. However, this database has many missing values since not all tests were administered in all studies. The best solution to handle this missing value problem is to fit a factor model to the data (Agelink van Rentergem, De Vent, Schmand, Murre, & Huizenga, 2018; Cudeck, 2000). However, this requires knowledge on the best-fitting factor model, which still needs to be determined.

Although domains have many uses, the idea of domains of cognitive functions is not without problems. There is a lack of consensus on which tests belong to which domain because there are many reasonable ways to assign tests to domains. For example, the Trail Making Test B (TMT B), in which one has to draw a line from labeled circles 1 to A to 2 to B to 3 and so on, is one test that is particularly difficult to assign. Because it involves drawing with a pencil and the outcome measure is the time to completion, one could assign it to the domain of psychomotor speed along with tests like the Grooved Pegboard. However, because Trail Making Test B performance depends for a large part on how attentive the person is, one could assign it to the domain of attention as well, along with tests like the Continuous Performance Test. Moreover, because it involves shifting back and forth between letters and numbers, one could assign it to the domain of executive functions along with the Stroop Interference test.

There is also a lack of consensus on how many domains there are. For example, there are many tests that aim to assess memory in neuropsychology. Whether a single memory domain is sufficient, or whether more domains are necessary, is a matter of debate (Delis, Jacobson, Bondi, Hamilton, & Salmon, 2003). Measures of memory could be divided into measures of an immediate recall domain and a delayed recall domain, or in measures of a visuospatial memory domain and a verbal memory domain. Of course, one could also argue that separate domains are necessary for immediate visuospatial recall and delayed visuospatial recall.

A factor analysis can provide some clarity through quantification of what model best describes the correlations between tests. We have up till now used the term domain interchangeably with the term latent variable. Domain is a more common term in clinical practice used to refer to a family of tests to which a particular test belongs. Latent variable is a more common term in research, used to refer to a variable that is measurable by inferring it from the correlations between test variables. Domain and latent variable are therefore not strictly synonymous. Because we perform a latent variable analysis, we use the term latent variable to be precise and consistent throughout the rest of the article. We will outline next how the latent variables that result from a latent variable analysis depend on the method and sample of the study.

First, the factor structure that is found will depend on the tests that are selected. For example, if a test like Trail Making Test B is administered together with tests that measure executive functioning, Trail Making Test B may also load on a single executive functioning factor because it has elements of shifting. However, if more speeded tests are administered, Trail Making Test B may load on a different latent variable, processing speed, together with other measures of processing speed. Therefore, the domain to which a test seems to belong is dependent on the battery of tests used. Consequently, comparisons across studies with different batteries of tests become necessary.

Second, age can affect the factor structure that is found in a study because age affects scores on almost all neuropsychological measures. Therefore, in a sample with a large age range, variables may become correlated because they are affected by age. Elderly people generally score lower on all variables, and young people generally score high on all variables. If age is not appropriately accounted for, fitting a factor model to a sample with a large age range can provide support for a single “cognitive” factor on which some participants score poorly - the elderly - and others score well - the young. One solution would be to study the factor structure in a sample that is homogeneous in age. However, since studying a single age group limits generalizability, an appropriate alternative is to include age in the analysis.

Third, similar to the age range effect, there can be a confounding effect of level of education in factor analysis. There is generally a large effect of education on neuropsychological test scores. Again, this may lead to the conclusion that to explain correlations between tests, we need just a single “cognitive” factor on which some participants score poorly - those with little education - and some score well - those with much education. Such a single factor due to education would not be found in samples with very similar educational background such as college students. However, since neuropsychological test results need to generalize beyond groups such as college students, it may again be more appropriate to correct for the effect of education in the analysis.

Fourth, domains can be different depending on the sample used. This is especially true for samples of patients with very specific deficits. A delayed recall test can become uncorrelated with other memory tests if delayed memory specifically is impaired by disorder or injury. Therefore, the structure of domains is ideally studied separately for healthy groups and different clinical groups. Results so far have shown that the factor structure has large communalities for many different clinical groups (Bowden, Cook, Bardenhagen, Shores, & Carstairs, 2004; Park et al., 2012; Schretlen et al., 2013), but it cannot be assumed that this is the case for all disorders.

Fifth, to obtain stable results for a factor analysis, many participants have to be tested on multiple tests. The amount of variance that is explained by latent variables may be low in neuropsychology, and examining many latent variables increases the required sample size (MacCallum, Widaman, Zhang, & Hong, 1999). However, obtaining a large sample size for a battery of neuropsychological tests is costly. This limits the number of participants that can be tested in a study, or limits the size of the battery that can be administered to a large number of participants.

Our goal is to establish how neuropsychological tests should be assigned to domains. We will do so by using a factor analytic approach, comparing different factor models that have been formulated in the literature. We will use the results of multiple studies to achieve a broad range of neuropsychological tests, and we will correct for effects of demographic variables including age and level of education. We will study healthy adults so the factor models are not confounded by sample differences in clinical status. Last, through combining different studies, samples of participants are combined to arrive at a much larger sample size than possible with a single study.

First, we will perform a factor analysis of neuropsychological tests by applying a meta-analytic framework that allows for structural equation models to be fitted to summary statistics (Cheung & Chan, 2005). Specifically, this method pools correlation matrices from multiple studies to arrive at a single correlation matrix. To this correlation matrix, multiple models can be fitted, which allows us to compare the fit of neuropsychological factor models that have been formulated in the literature. Second, we will conduct a factor analysis of data from the ANDI normative database (De Vent et al., 2016a). This database contains raw data from healthy control participants from multiple studies conducted in the Netherlands and Belgium, not included in our first analysis.

To summarize, neuropsychology would benefit from clarity on the number and type of cognitive domains and on which tests belong to which cognitive domains. This would facilitate test selection, diagnosis of single-domain and multi-domain disorders, calculation of composite scores, neuropsychological research into the construct validity of tests, and normative comparisons given the aggregated ANDI database.

## Study 1: Factor Meta-Analysis

### Methods

#### Literature Search

A systematic literature search was conducted using PsycINFO and MEDLINE for articles that contained a factor analysis of neuropsychological tests in healthy adults. Factor analyses were chosen since studies conducting a factor analysis generally recruit a large sample and administer a large battery of tests. The search strategy was developed in PsycINFO (see Appendix 1 for the syntax) because PsycINFO is particularly well-suited for searching psychological tests. The search strategy for MEDLINE was based on the PsycINFO search strategy. The search strategy consisted of the following key concepts: factor analysis-related terms, specific neuropsychological test-related terms and general neuropsychology-related terms. Deduplication of results was done using *Refworks*, and screening of results for inclusion was done using *Rayyan* (Ouzzani, Hammady, Fedorowicz, & Elmagarmid, 2016).

##### Exclusion Criteria

The goal was to obtain a healthy adult sample correlation matrix from each study containing both neuropsychological tests and demographic variables. Articles were excluded if a) fewer than two tests of interest were used, b) an adult sample was not studied, c) they were published before 1997, d) a cognitively healthy sample was not studied, e) test administration was manipulated or otherwise differed from typical administration, f) they were included in the ANDI database. Criterion c was chosen because for many datasets, extra information would be required from the original authors. Criterion d entailed that we did not include groups with psychiatric or neurological disorders (e.g., bipolar disorder or epilepsy), with disorders that could interfere with test administration (e.g., hearing loss), or with conditions that were studied for their cognitive implications (e.g., HIV). Criterion e excluded studies in which manipulations (e.g., transcranial magnetic stimulation) were applied to participants during testing, or in which novel, often computerized, versions of test batteries were used. This last choice was made because these novel versions are less familiar and less thoroughly validated than the common versions. Criterion f preserved the independence of the analyses done in study 1 and study 2 of the present article.

##### Tests

A complete list of variables that were considered of interest is given in Appendix 2. We included search terms for each of these variables. We intended to collect correlations from several tests from each of the models’ domains. However, far from every combination of variables was present in the correlation matrices that were analyzed. Therefore, even though we included search terms for, for example, Rey Complex Figure Test, Judgement of Line Orientation, Tower of London, and Wisconsin Card Sorting Test, and we requested correlations for all these tests, there was no complete overlap with all other common test variables for these tests. For twelve test variables, correlations were available with every other variable. To increase the number of usable correlations, different versions of the same test variables were combined (see Table 1). These tests may not be completely parallel since there may be differences in test administration and scoring rules. However, the current analysis assumes that, although there may be mean differences between versions, the correlations with other test variables will not be different. This issue is addressed in study 2.

**Table 1:** Included test variables

Test variable	Abbreviation	Additional information
Trail Making Test Part A	TMTA	Combined with Color Trails Test Part 1, D-KEFS Trail Making Test condition 2.
Trail Making Test Part B	TMTB	Combined with Color Trails Test Part 2, D-KEFS Trail Making Test condition 4.
Story Recall Immediate Recall	SR-IR	Combined across multiple WMS Logical Memory versions, combined with RBANS Story Immediate Memory.
Story Recall Delayed Recall	SR-DR	Combined across multiple WMS Logical Memory versions, combined with RBANS Story Delayed Memory.
Letter Fluency	LF	Synonyms: Controlled Oral Word Association Test, Phonemic Verbal Fluency.
Semantic Fluency	SF	Synonyms: Categorical Verbal Fluency. Preferential inclusion of the “Animals” version if multiple were available.
Digit Span Forwards	DSF	Combined across multiple WAIS and WMS versions.
Digit Span Backwards	DSB	Combined across multiple WAIS and WMS versions.
Coding	COD	Combined across multiple WAIS versions. Synonym: Digit Symbol Substitution.
Boston Naming Test	BNT	
Auditory Verbal Learning Test – Total Recall	VLT-TR	Combined with California Verbal Learning Test – Total Recall, the Hopkins Verbal Learning Test – Total Recall, and RBANS List Learning.
Auditory Verbal Learning Test – Delayed Recall	VLT-DR	Combined with California Verbal Learning Test – Long-Delay Recall, the Hopkins Verbal Learning Test – Delayed Recall, and RBANS List Recall.

##### Contacting Authors

With a few exceptions (e.g., Adrover-Roig, Sesé, Barceló, & Palmer, 2012), articles or supplementary materials did not contain the correlation matrix including both the tests and the demographic variables that were necessary for this study. Therefore, corresponding authors of included studies were contacted. In case a researcher appeared multiple times as a corresponding author in the included studies, a single, recent article was chosen which included a large selection of tests. In this case, if the corresponding authors agreed to share a correlation matrix, they were asked whether they would be willing to share the correlation matrix for other articles as well. If authors did not respond, they were reminded after a period of 2–3 weeks.

The authors were sent a list of variables of interest that were to be included, which were the test variables that they collected in their study along with age, sex, and level of education. There was no specific hypothesis for the influence of sex on the factor structure, but we chose to correct for its influence as well because this is common in neuropsychology (Testa, Winicki, Pearlson, Gordon, & Schretlen, 2009). Level of education was scored differently in different studies, sometimes using a seven-point-scale, sometimes using years of education. This issue is discussed in more depth in the discussion section and is addressed in study 2. Authors were requested to send a correlation matrix of these variables for the cognitively healthy sample within their data. If they were unsure that their participants qualified as cognitively healthy, possibilities for exclusion criteria within their data were discussed. For example, if measurements from the Mini-Mental State Examination (MMSE; Folstein, Folstein, & McHugh, 1975) and Clinical Dementia Rating (CDR; Morris, 1993) had been taken in their study, participants with MMSE scores below 24 and CDR scores above 0 could be removed before the correlation matrix was computed. Since these exclusion criteria depended on what the authors had available in their data, this was an ad-hoc procedure.

Publication bias was not considered. Publication bias exists when the publication and subsequent inclusion of studies is contingent on the size of the effect that is of interest in the meta-analysis (Cheung & Vijayakumar, 2016; Easterbrook, Gopalan, Berlin, & Matthews, 1991). The risk of publication bias is negligible because the information that is retrieved are correlations rather than effect sizes of group comparisons.

##### Analysis

The analysis was carried out using *R* (R Core Team, 2016). First, for each study, the correlation matrix was converted to a partial correlation matrix by partialing out the influence of age, sex, and level of education using the *psych* package (Revelle, 2008). This method allows for study-specific correction for age, sex, and level of education.

A factor meta-analysis of the partial correlation matrices was conducted using the *metaSEM* package (Cheung, 2015, see Electronic Supplementary Material 2 for CHC example script). This factor meta-analysis consisted of two steps (Cheung & Chan, 2005; Jak, 2015). First, the partial correlation matrices were pooled into a single weighted partial correlation matrix using the total number of participants after exclusion for each study in the weighting. The number of participants per study was recorded as the lowest number of participants that were available for any given correlation within the study since sample sizes may differ due to missing data. Therefore, the total number of participants included here is an underestimate of the actual number of participants, and the confidence intervals could in fact be tighter for some correlations. Second, using the weighted partial correlation matrix as input, different factor models that have been described in the literature were compared (Lee, 1986; Levin, 1987; McDonald, 1978). For each model, fit was evaluated by RMSEA, SRMR, CFI, AIC, and BIC, using the rules of thumb outlined in Schermelleh-Engel, Moosbrugger, and Müller (2003) to decide what constitutes lack of fit, acceptable and good fit.

##### Candidate Factor Models

Factor models that were broad enough to span all neuropsychological tests were selected from the literature. This excludes factor models that describe correlations between tests from just a single domain (e.g., Huizinga, Dolan, & van der Molen, 2006). The first model was a model with a single latent variable on which all variables loaded. Verhaeghen and Salthouse (1997) used a single factor model in a meta-analysis of correlations of neuropsychological test scores and found that a large part of the variance in test scores can be construed as variance on a single common latent variable. The fit of the one factor model can be used as a reference to judge the fit of more complex models.

The second and third models came from the chapter structure of the clinical neuropsychology reference works by Strauss, Sherman, and Spreen (2006) and Lezak, Howieson, Bigler, and Tranel (2012). Although there is not an explicit factor model in these works that has been empirically tested, the neuropsychological tests are categorized into separate chapters. Therefore, they give a good impression of which tests belong together in the eyes of clinical neuropsychologists. In Strauss, Sherman, and Spreen (2006), the chapters containing the included tests were “General cognitive functioning”, “Executive Functions,” “Memory, “Orientation and attention,“ and “Language“. In Lezak, Howieson, Bigler, and Tranel (2012), the chapters containing the included tests were “Attention,” “Memory,” “Executive Functions,” “Verbal functions and language skills.“ The difference between the two was that Digit Span and Coding fall under “General cognitive functioning“ in Strauss, Sherman, and Spreen (2006) and under “Orientation and attention” in Lezak, Howieson, Bigler, and Tranel (2012).

The fourth and fifth models were based on the opinion of experts. The fourth model was based on the domains used in Gross et al. (2015). Gross et al. (2015) assigned tests to “Memory”, “Executive functioning” and “Rest” domains on the basis of expert opinion. Of the currently included tests, only the Boston Naming Test fell in the “Rest” category. The fifth model was based on a survey of clinical neuropsychologists (Hoogland et al., 2017). Twenty experts were asked to rate, on a seven-point Likert scale, how well test variables assess cognitive functioning on a particular domain. For the twelve tests included here, the relevant domains were “Language,” “Attention and working memory,” “Memory,” and “Executive function.” For the factor model used, all mean ratings were above 4.85 on the seven-point scale indicating a large degree of confidence that these variables should be assigned to these domains.

The sixth model was based on the recommendations made by Larrabee (2014). Larrabee (2014) divided tests in six domains on the basis of a review of the literature. This domain specification was explicitly intended to help clinicians compose a battery of tests that assesses cognitive abilities from different domains. The four domains for the included tests are “Verbal symbolic abilities,” “Attention or working memory,” “Processing speed,” and “Learning and memory—verbal and visual.”

The seventh and eighth models were two variants of the Cattell-Horn-Carroll (CHC) model as described by Jewsbury, Bowden, and Duff (2016). The CHC model was developed in intelligence research rather than in clinical neuropsychology (Floyd, Bergeron, Hamilton, & Parra, 2010; McGrew, 2009; Schneider & McGrew, 2018). Although the CHC model is dominant and has a broad influence on the development of IQ test, there is not one single CHC model and the CHC model has developed over time. The CHC model started as a combination of the three-stratum Carroll model, which includes a higher-order factor g, with the Cattell-Horn model, which splits cognitive functioning into fluid and crystallized parts, Gf and Gc (McGrew, 2009; Schneider & McGrew, 2018). Since then, the CHC was adapted many times and now broadly includes nine different factors (Keith & Reynolds, 2010; McGrew, 2009; Schneider & McGrew, 2018). For a review of how the Cattell-Horn-Carroll model came into existence, and how it has evolved over time, see Schneider and McGrew (2018). Jewsbury, Bowden, and Duff (2016) formulated one specific version of this CHC model where the higher-order factor g is not included. Therefore, it can be argued that their model does not include Carroll’s original contribution. However, in accordance with Jewsbury et al., we still characterize this version as the CHC model. Jewsbury, Bowden, and Duff (2016) demonstrated that the CHC model as they formulate it fits well in each of the nine neuropsychological datasets they studied, with only minor adaptations for each dataset. One addition to the CHC, as it was adapted to clinical neuropsychology, was the inclusion of Fluency as a separate latent variable (Jewsbury & Bowden, 2016; Schneider & McGrew, 2018).

The factors for the included tests were the same across the two variants of the CHC model: “Acquired knowledge or crystallized ability,” “Processing speed,, “Long-term memory encoding and retrieval,” “Working memory,“ and “Word fluency,. In the first variant, Trail Making Test B measures “Processing speed.” In the second variant, Trail Making Test B measures both “Processing speed” and “Working memory.”

All factor model specifications are given in Table 2. As factor names, we have chosen to follow the naming conventions from the sources of the different factor models. Because we could not include all variables that were included in the original models, some other factor names might be more apt. For example, the factor “Long term memory encoding and retrieval” from the CHC model might also be called “Memory” as the tests that fall under this factor in the CHC model are almost the same as those that fall under the factor called “Memory” in other models.

**Table 2:** Factor model specifications of the candidate models for study 1. Tests that load on the same latent variable share a letter. Some tests load on multiple latent variables in the Hoogland and CHC models

	TMTA	TMTB	SR-IR	SR-DR	LF	SF	DSF	DSB	COD	BNT	VLT-TR	VLT-DR
One factor (Verhaeghen & Salthouse, 1997)	A	A	A	A	A	A	A	A	A	A	A	A
Strauss, Sherman, and Spreen (2006)	D	D	C	C	B	B	A	A	A	E	C	C
Lezak, Howieson, Bigler, and Tranel (2012)	A	A	B	B	C	C	A	A	A	D	B	B
Gross et al. (2015)	A	A	B	B	A	A	A	A	A	C	B	B
Hoogland et al. (2017)	B	B + D	C	C	A+ D	A + D	B	B	B	A	C	C
Larrabee (2014)	A	A	B	B	C	C	D	D	A	C	B	B
CHC 1 (Schneider & McGrew, 2018)	B	B	A + C	A + C	E	E	D	D	B	A	C	C
CHC 2 (Schneider & McGrew, 2018)	B	B + D	A + C	A + C	E	E	D	D	B	A	C	C

*TMTA* Trail Making Test A, *TMTB* Trail Making Test B, *SR-IR* Story Recall Immediate Recall, *SR-DR* Story Recall Delayed Recall, *LF* Letter Fluency, *SF* Semantic Fluency, *DSF* Digit Span Forwards, *DSB* Digit Span Backwards, *COD* Digit Symbol Substitution or Coding, *BNT* Boston Naming Test, *VLT-TR* Verbal Learning Test - Total Recall, *VLT-DR* Verbal Learning Test - Delayed Recall, *CHC* Cattell-Horn Carroll

Each factor model consisted of factor loadings describing the relationship between the tests and the latent variables, residual variances of the test variables, and covariances between latent variables, which were all freely estimated. The covariances between latent variables can be interpreted as correlations, because all latent variable variances were fixed to 1.

## Results

### Sample

From the literature search, 3259 sources were identified. After deduplication, 2520 distinct sources remained. These were judged against the exclusion criteria by inspection of the title, abstract, and description of the tests and measures that is provided in PsycINFO. After this step, 330 articles were selected of which the full-texts were obtained. Seven articles were excluded because the full-text could not be could not be retrieved, so a total of 323 were eligible for inclusion. After e-mailing the corresponding authors, 60 correlation matrices were obtained from 57 studies. A list of contributing studies is provided in the supplemental material. Horvat et al. (2014) provided four separate correlation matrices from four countries.

**Fig. 1:**
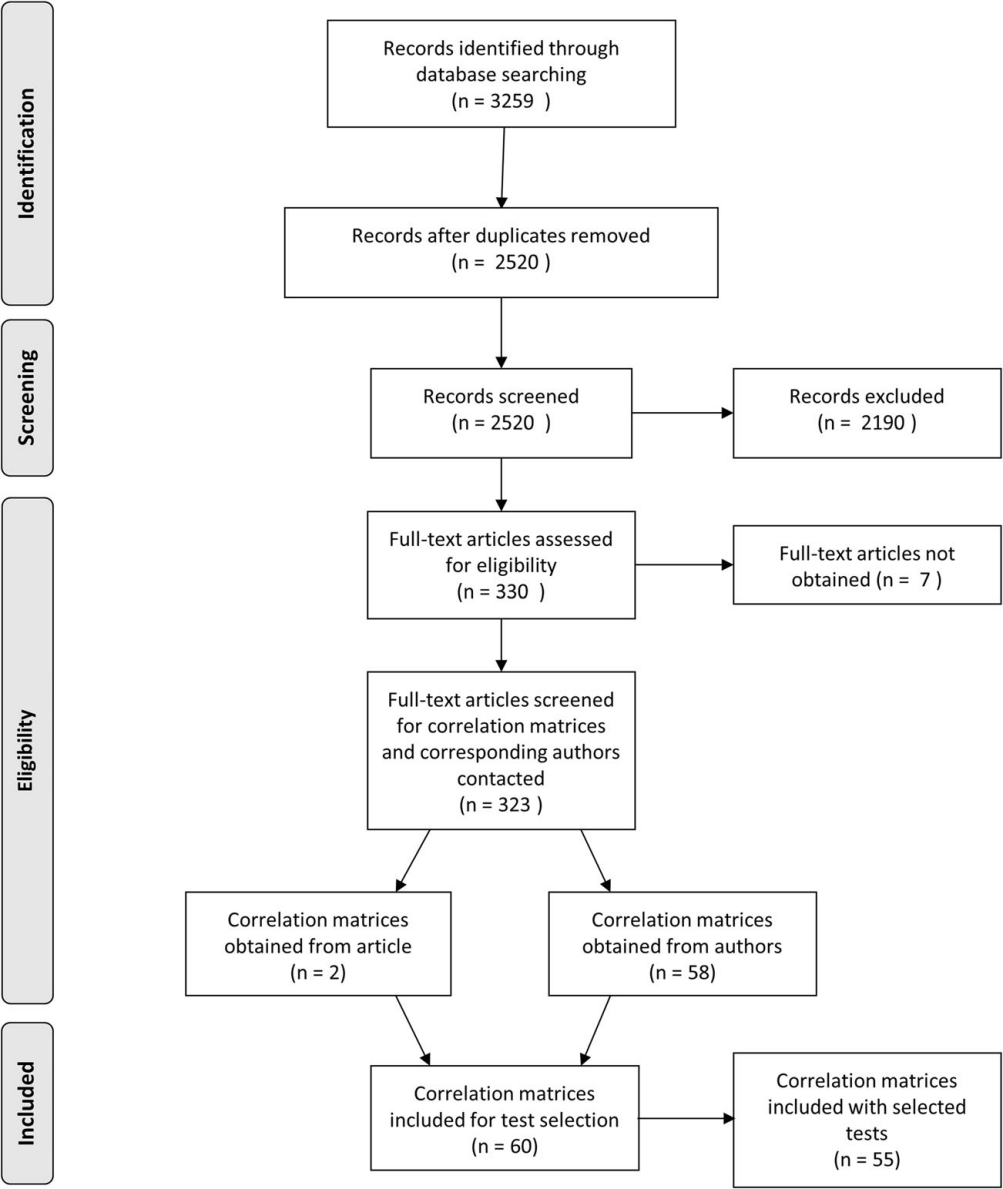
PRISMA diagram

From these correlation matrices, tests were selected that were administered together in multiple studies. This limited the number of tests to the twelve described in the methods section. Five studies did not include any or just one of the selected tests and were not included in the final analysis (Burns, Nettelbeck, & McPherson, 2009; DeYoung, Peterson, & Higgins, 2005; Kafadar, 2012; Sternäng, Lövdén, Kabir, Hamadani, & Wahlin, 2016; Thibeau, McFall, Wiebe, Anstey, & Dixon, 2016). The PRISMA diagram is given in Fig. 1.

All correlations of test variables were scrutinized for miscoding. One source showed aberrant correlations that could not be explained because Trail Making Test B was positively correlated with other, unspeeded, tests in one correlation matrix (the oddity of which was noted in the original publication; Royall, Bishnoi, & Palmer, 2015). Correlations with the Trail Making Test B variable were removed for this study. Motivating plots for this removal are provided in Appendix 3, along with the analysis which did include these correlations.

The final sample consisted of 60,398 participants and 55 correlation matrices. Study characteristics are given in Appendix 4, along with those correlation matrices for which we received explicit permission to share them here (49 out of 55). The correlations with age, sex, and level of education were partialed out from each correlation matrix. Because for some correlations, multiple studies provided data, we attempted to estimate the variability between studies in these correlations by introducing one or more variance components to the model. However, this led to a lack of convergence of the first part of the analysis, establishing the pooled weighted partial correlation matrix, at every attempt. Therefore, the pooled weighted partial correlation matrix was established using a fixed meta-analysis approach. The pooled partial correlation matrix is given in Table 3.

**Table 3:** Pooled partial correlation matrix

	TMTA	TMTB	SR-IR	SR-DR	LF	SF	DSF	DSB	COD	BNT	VLT-TR
TMTB	0.543 *(0.006)*										
SR-IR	−0.084 *(0.012)*	−0.171 *(0.011)*									
SR-DR	−0.091 *(0.012)*	−0.176 *(0.011)*	0.864 *(0.002)*								
LF	−0.207 *(0.009)*	−0.264 *(0.009)*	0.198 *(0.015)*	0.208 *(0.014)*							
SF	−0.222 *(0.009)*	−0.256 *(0.008)*	0.262 *(0.009)*	0.274 *(0.009)*	0.457 *(0.006)*						
DSF	−0.117 *(0.010)*	−0.202 *(0.010)*	0.151 *(0.010)*	0.134 *(0.010)*	0.231 *(0.011)*	0.168 *(0.009)*					
DSB	−0.159 *(0.011)*	−0.283 *(0.010)*	0.236 *(0.010)*	0.220 *(0.011)*	0.272 *(0.011)*	0.230 *(0.009)*	0.481 *(0.007)*				
COD	−0.487 *(0.012)*	−0.516 *(0.012)*	0.239 *(0.011)*	0.256 *(0.011)*	0.351 *(0.012)*	0.348 *(0.008)*	0.187 *(0.010)*	0.271 *(0.010)*			
BNT	−0.185 *(0.013)*	−0.195 *(0.012)*	0.258 *(0.010)*	0.267 *(0.010)*	0.234 *(0.011)*	0.284 *(0.008)*	0.128 *(0.012)*	0.153 *(0.014)*	0.304 *(0.012)*		
VLT-TR	−0.154 *(0.017)*	−0.217 *(0.018)*	0.447 *(0.010)*	0.461 (0.009)	0.267 *(0.018)*	0.349 *(0.004)*	0.196 *(0.011)*	0.271 *(0.012)*	0.300 *(0.011)*	0.232 *(0.010)*	
VLT-DR	−0.140 *(0.018)*	−0.154 *(0.018)*	0.439 *(0.010)*	0.502 (0.009)	0.197 *(0.022)*	0.322 *(0.008)*	0.092 *(0.012)*	0.183 *(0.012)*	0.278 *(0.012)*	0.225 *(0.010)*	0.695 *(0.005)*

In Parentheses (Italics): Standard Errors of Estimated Mean Correlations According to the Fixed Effects Model

*TMTA* Trail Making Test A, *TMTB* Trail Making Test B, *SR-IR* Story Recall Immediate Recall, *SR-DR* Story Recall Delayed Recall, *LF* Letter Fluency, *SF* Semantic Fluency, *DSF* Digit Span Forwards, *DSB* Digit Span Backwards, *COD* Digit Symbol Substitution or Coding, *BNT* Boston Naming Test, *VLT-TR* Verbal Learning Test - Total Recall, *VLT-DR* Verbal Learning Test - Delayed Recall

### Model Fit

The results of the model comparison between candidate models is given in Table 4. The Hoogland et al. (2017), Lezak, Howieson, Bigler, and Tranel (2012), and Strauss, Sherman, and Spreen (2006) models did not converge. Therefore, the fit measures for these models cannot be interpreted with confidence and are not reported. With respect to relative fit, the AIC and BIC indicate that the two variants of the CHC model fit better than the other models.

**Table 4:** Model comparison results

	RMSEA	SRMR	CFI	AIC	BIC
Hoogland et al. (2017)*	–	–	–	–	–
Lezak, Howieson, Bigler, and Tranel (2012)*	–	–	–	–	–
Strauss, Sherman, and Spreen (2006)*	–	–	–	–	–
One factor (Verhaeghen & Salthouse, 1997)	0.056	0.218	0.941	10,303.2	9816.8
Gross et al. (2015)	0.045	0.145	0.965	6084.2	5624.8
Larrabee (2014)	0.031	0.098	0.984	2735.5	2303.1
CHC 1 (Schneider & McGrew, 2018)	0.023	0.060	0.993	1250.0	871.7
CHC 2 (Schneider & McGrew, 2018)	0.022	0.060	0.993	1207.5	838.2

*Model did not converge, CHC = Cattell-Horn Carroll

With respect to absolute fit, the fit measures generally agree about the ordering of the models as well. All RMSEA values indicate good fit (all RMSEA <0.05), except for the one factor model, where the RMSEA value indicates acceptable fit (RMSEA <0.08). The SRMR values indicate a lack of fit for the five simplest models (SRMR >0.10), and acceptable fit for the CHC model and the model by Larrabee (SRMR >0.05). The CFI values indicate a lack of fit for the one factor model (CFI < 0.95), acceptable fit for the model used by Gross et al. (CFI < 0.97), and good fit for the other models (CFI > 0.97).

**Fig. 2:**
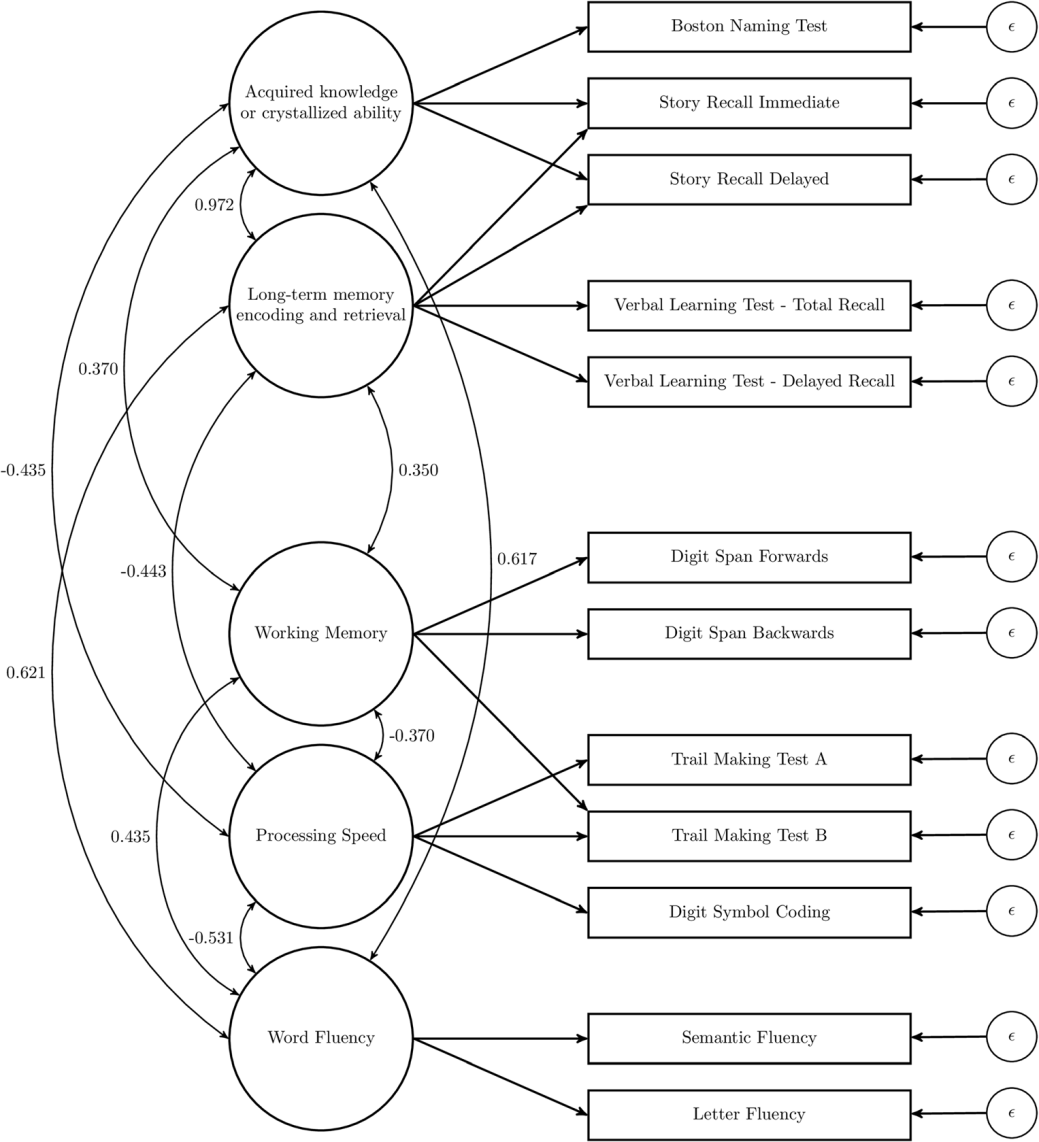
CHC 2 model for the twelve tests included in study 1. For each combination of latent variables, the correlation is given

The best-fitting CHC model is depicted in Fig. 2 in which correlations between latent variables are also provided. Because Trail Making Test A and B are measured in time to completion, these variables and the “Processing Speed” factor that they loaded on, are reverse coded. Therefore, the negative correlations between “Processing Speed” and the other latent variables should be interpreted such that better “Processing Speed” is correlated with better scores on the other latent variables.

## Discussion

From this factor meta-analysis, we can conclude that the two CHC models provide the best fit. The two CHC models themselves do not differ by much, but all fit measures agree that the second model, with the extra cross-loading of Trail Making Test B on “Working memory,” fits better.

Therefore, we conclude that for the tests used here, the correlations between test variables are best described by five cognitive domains, namely “Acquired knowledge or crystallized ability,” “Processing speed,” “Long-term memory encoding and retrieval,” “Working memory,” and “Word fluency.” We also conclude that some test variables load on multiple domains.

The factor meta-analysis framework has several advantages in that it allows for the analysis of a large number of tests and a very large number of participants. Using the partial correlation matrices rather than the raw correlation matrices allowed us to correct for the effects of age, sex, and level of education.

However, there are a number of limitations to this analysis. First, different versions of tests were used as if they are parallel. This choice was made to arrive at a greater degree of test overlap between studies. Whether a test with a different name constitutes a different version or a different test altogether is to some degree subjective. For some tests, there is empirical evidence that there is a high correlation between test scores which makes it reasonable for the present goal to consider them versions of the same test. For example, the sum scores of the California Verbal Learning Test (CVLT), the Hopkins Verbal Learning Test (HVLT), and the Auditory Verbal Learning Test (AVLT) are highly correlated (for example, Lacritz & Cullum, 1998, report r = 0.74 between CVLT and HVLT; Stallings, Boake, & Sherer, 1995, report r = 0.83 between CVLT and AVLT; an anonymous reviewer reports r = 0.68 between HVLT and AVLT), even though there are differences between test versions in stimuli, test administration, the number of repetitions, and so on. For the Color Trails and Trail Making Test, the pooling of data may be more contentious since the tests are more dissimilar (but see Dugbartey, Townes, & Mahurin, 2000; Lee & Chan, 2000). The assumption here was that the correlations between these variables and other test variables do not change due to these differences. This assumption may not be tenable.

Second, there were differences in education scales and education systems between studies. As argued in the introduction, it is necessary to remove the confounding influence of education. However, the contributing studies used different ways of coding level of education, which means that the correction in the form of the partial correlation was different between studies as well. Also, even if two studies used the same scale such as years of education, such a scale may have a different interpretation in different countries (UNESCO, 2011; De Vent et al., 2016b).

Third, there was some overlap in the studies that were used in Jewsbury, Bowden, and Duff (2016) and the studies that were included in this factor meta-analysis. Thus, the sample that was used to develop the model was not completely distinct from the sample used to evaluate its performance. To study whether the results were biased towards the model as specified by Jewsbury, Bowden, and Duff (2016), we reran the analysis without two datasets that were included in their analysis (Duff et al., 2006; Bowden, Cook, Bardenhagen, Shores, & Carstairs, 2004). Without these studies, the Gross, CHC 1, and CHC 2 models did not converge (in addition to Hoogland and Lezak that did not converge before). Of the remaining models (Strauss, One factor, and Larrabee), the Larrabee model fitted best. The two excluded studies had a wide range of tests as well as a large sample, and therefore played an important part in stabilizing results. The analysis presented here and in Jewsbury, Bowden, and Duff (2016) were therefore not independent, which could have artificially improved the performance of the CHC model.

To address these issues, in the next study, the factor models will be fitted to raw data from the Netherlands and Belgium, combined in the ANDI database. This database allows us to use a single test version for every variable, and to use a single standardized education scale. Also, because raw data are available, we can directly incorporate the influence of demographic variables on test variables, rather than using the more indirect approach of partialing out these variables from the correlations. Last, this is a completely different sample of studies from the samples used in study 1 and the samples used by Jewsbury, Bowden, and Duff (2016).

### Study 2: Factor Analysis of the ANDI Database

#### Methods

##### Sample

The construction and composition of the ANDI database are described elsewhere (De Vent et al., 2016a). This database includes data of studies that were conducted in the Netherlands and Belgium. For the data used in the present analysis, the number of included studies was 54 with a total of 11,881 participants. In Study 2, Full Information Maximum Likelihood (FIML) was used for model estimation. FIML allows for estimation with missing data and allows us to estimate covariances between tests that are implied by the factor analysis, even when pairs of variables are missing (Agelink van Rentergem, De Vent, Schmand, Murre, & Huizenga, 2018; Cudeck, 2000). This was not possible for Study 1 where raw data were not available. In Study 2, we were able to estimate almost the exact same model with a much smaller number of participants and a smaller number of studies than in Study 1. Without using FIML to deal with missing data in the variables, fewer variables would be amenable for inclusion in Study 2.

All test variables were transformed to normality using Box-Cox transformations (Box & Cox, 1964, selected power transformations are reported on andi.nl/en) in order to meet parametric assumptions and to speed up convergence, and were demographically corrected and standardized (De Vent et al., 2016a). Note that factor analyses can also be performed on non-normally distributed data, but in the ANDI project it was required to transform data to normality. For the demographic corrections for level of education, we used a seven-point scale that is commonly used in Dutch neuropsychology (Verhage, 1964). This scale is comparable to the International Standard Classification of Education (UNESCO, 2011).

##### Tests

In study 2, the same test variables were included as in study 1. To remove the influence of test versions differing between studies, we included a single version for every test. Digit Span Forwards and Backwards were not included since there were too few data for these variables for any specific version. Story Recall Immediate Recall and Story Recall Delayed Recall referred to Rivermead Behavioural Memory Test Stories Immediate Recall and Delayed Recall. Semantic Fluency referred to the Animals version of Semantic Fluency. Coding referred to WAIS-III Digit Symbol-Coding. Verbal Learning Test referred to the Rey Auditory Verbal Learning Test.

##### Model Changes

Because of the removal of the Digit Span subtests, the two versions of the CHC model collapse into a single version without “Working memory.” The remaining factors were “Acquired knowledge or crystallized ability,” “Processing speed,” “Long-term memory encoding and retrieval,” and “Word fluency.” Like in study 1, factor loadings and covariances between latent variables were freely estimated. All latent variable variances were fixed to 1 so the covariances between latent variables can be interpreted as correlations. Residual variances of the tests are freely estimated as well. For three models, there was an exception because these models included factors with a single indicator (Strauss, Lezak, and Gross) (Table 5). For these single indicators, factor loadings were constrained to equal 1 and residual variances were constrained to equal 0.

**Table 5:** Factor model specifications of the candidate models for Study 2

	TMTA	TMTB	SR-IR	SR-DR	LF	SF	COD	BNT	VLT-TR	VLT-DR
One factor (Verhaeghen & Salthouse, 1997)	A	A	A	A	A	A	A	A	A	A
Strauss, Sherman, and Spreen (2006)	D	D	C	C	B	B	A	E	C	C
Lezak, Howieson, Bigler, and Tranel (2012)	A	A	B	B	C	C	A	D	B	B
Gross et al. (2015)	A	A	B	B	A	A	A	C	B	B
Hoogland et al. (2017)	B	B + D	C	C	A + D	A + D	B	A	C	C
Larrabee et al. (2014)	A	A	B	B	C	C	A	C	B	B
CHC (Schneider & McGrew, 2018)	B	B	A + C	A + C	E	E	B	A	C	C

Tests that Load on the Same Latent Variable Share a Letter. Some Tests Load on Multiple Latent Variables in the Hoogland and CHC Models

*TMTA* Trail Making Test A, *TMTB* Trail Making Test B, *SR-IR* Story Recall Immediate Recall, *SR-DR* Story Recall Delayed Recall, *LF* Letter Fluency, *SF* Semantic Fluency, *COD* Digit Symbol Substitution or Coding, *BNT* Boston Naming Test, *VLT-TR* Verbal Learning Test - Total Recall, *VLT-DR* Verbal Learning Test - Delayed Recall, *CHC* Cattell-Horn Carroll

The models were fitted using Mplus (Muthén & Muthén, 2012; see Electronic Supplementary Material 2 for CHC example input). Like in Study 1, we attempted to include variance components within the model since the data are nested within studies and are not strictly independent. The standard approach is to include the different levels within the analysis, estimating a variance component at the different levels in a multilevel SEM model (Hox, Maas, & Brinkhuis, 2010). However, when including variance components, none of the models converged, presumably due to the high percentage of missing data within the ANDI database (De Vent et al., 2016a). Therefore, variance components were not included for the results given below.

Fit was evaluated by RMSEA, SRMR, CFI, AIC, and BIC using the rules of thumb outlined in Schermelleh-Engel, Moosbrugger, and Müller (2003) to decide what constitutes lack of fit, acceptable, and good fit.

## Results

The Strauss model did not converge. The CHC model converged, but produced a warning indicating a negative residual variance which may indicate misspecification if the negative variance is large (Kolenikov & Bollen, 2012). However, the variance was not significantly different from 0, θ = −0.032, z = −0.581, *p* = 0.561.

The results of the model comparison between candidate models is given in Table 6. As in study 1, the fit measures for the model that did not converge could not be interpreted and were not reported. With respect to relative fit, the AIC and BIC indicate that the CHC model fits better than the other models.

**Table 6:** Model comparison results

	RMSEA	SRMR	CFI	AIC	BIC
Strauss, Sherman, and Spreen (2006)*	–	–	–	–	–
One factor (Verhaeghen & Salthouse, 1997)	0.065	0.149	0.736	73,659.1	73,880.6
Gross et al. (2015)	0.040	0.111	0.904	72,634.4	72,863.3
Hoogland et al. (2017)	0.035	0.103	0.942	72,407.6	72,688.1
Lezak, Howieson, Bigler, and Tranel (2012)	0.032	0.092	0.944	72,388.4	72,639.5
Larrabee (2014)	0.031	0.095	0.944	72,388.6	72,632.3
CHC (Schneider & McGrew, 2018)	0.013	0.054	0.992	72,098.1	72,378.7

*Model did not converge or produced an error. - = not reported due to lack of convergence

All RMSEA values indicate good fit (all RMSEA <0.05), except for the one factor model, for which the RMSEA indicates acceptable fit (RMSEA <0.08). The SRMR values indicate a lack of fit for the one factor, Gross, and Hoogland models (SRMR >0.10), and acceptable fit for the Lezak, Larrabee, and CHC models (SRMR >0.05). The CFI values indicate a lack of fit for all models (CFI < 0.95), except for the CHC model, for which fit was good (CFI > 0.97).

**Fig. 3:**
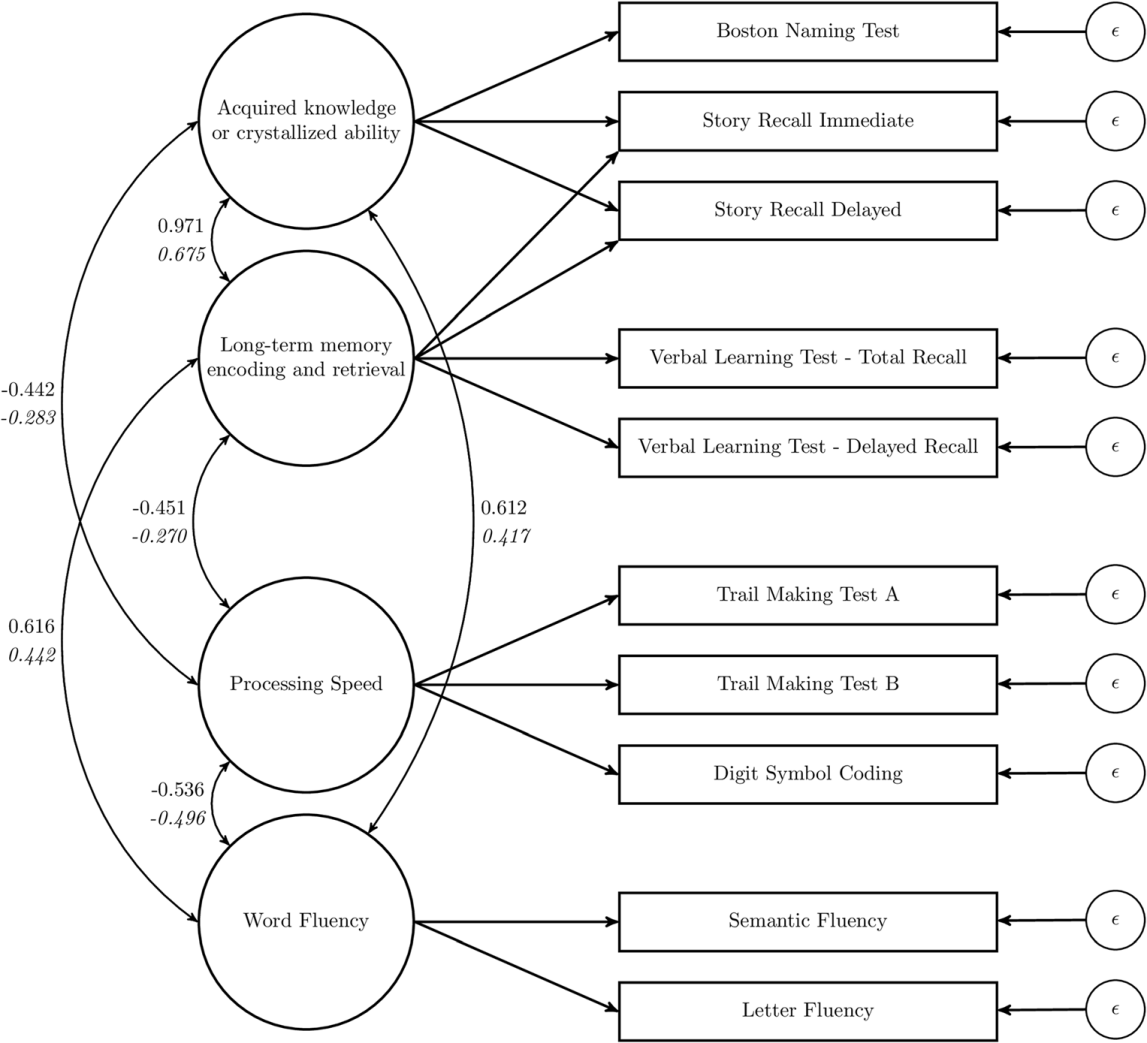
CHC model for the ten tests included in study 2. For each combination of latent variables, the correlation is given for the meta-analytic data in roman type, and for the ANDI data in italic type

Next, we compared the CHC model fitted in study 2 to the CHC model fitted in study 1 to determine whether the factor structure was stable across the two analyses. The methods used in the two studies were dissimilar, that is, correlation matrices served as the outcome measure in study 1 and actual test scores were the outcome measure in study 2. Because the scale of factor loadings and residual variances is dependent on the scale of the outcome measure, it is not warranted to compare factor loadings or residual variances between studies. However, the correlations between latent variables can be compared. To make the models comparable, the CHC model without the “Working Memory” latent variable from study 2 was fitted to the meta-analytic data from study 1 without Digit Span Forwards and Digit Span Backwards. The model is depicted in Fig. 3 in which correlations between latent variables are also provided. As in study 1, the “Processing Speed” factor is reverse coded. It can be seen that the correlations were in the same direction in both studies and that correlations were lower for the second study. This could be due to the more appropriate demographic corrections since regression-based corrections of the raw data were used rather than using a partial correlation approach, and level of education was coded on the same seven-point scale for all included samples.

## General Discussion

In this article, we sought to establish the cognitive domains that are measured by neuropsychological tests. Cognitive domains are used by neuropsychologists to make decisions on which tests to administer to a particular patient, to determine whether a disorder affects a single domain or multiple domains, to calculate composite scores of different tests belonging to the same domain, and to validate new tests that are designed to measure a particular cognitive function.

We compared several neuropsychological factor models that have been formulated in the literature. First, we performed a factor meta-analysis of correlation matrices, using the meta-analytic structural equation modeling framework (Cheung & Chan, 2005). Second, the different factor models were fitted to raw data from the ANDI database (De Vent et al., 2016a). Both analyses included a number of neuropsychological tests, a very large sample, and accounted for the effects of age, sex, and level of education. Using these two different methods and samples, the same result was obtained. The Cattell-Horn-Carroll (CHC) model was shown to be the model that best described the data.

In the introduction, we formulated the aims of the study to decide how many domains exist, what these domains are, and which cognitive tests belong to which domain. In this article, we were interested to examine the factor structure of all common neuropsychological test variables but were restricted by the availability of data to the twelve most frequently used test variable. From the factor analysis, we can conclude that the CHC model is an appropriate way of categorization for these twelve variables. For the tests that were considered in this article, the CHC model consists of five intercorrelated factors: “Acquired knowledge or crystallized ability”, “Long-term memory encoding and retrieval”, “Processing speed”, “Working memory”, and “Word fluency”. The Boston Naming Test and Story Recall variables loaded on the first factor. The Verbal Learning Test variables and Story Recall variables loaded on the second factor. Digit Symbol Substitution and Trail Making Test Parts A and B loaded on the third factor. The Digit Span variables and Trail Making Test Part B loaded on the fourth factor. Letter Fluency and Semantic Fluency loaded on the fifth factor.

The CHC model has three unique aspects compared to the other models fitted in this article. First, Letter Fluency and Semantic Fluency are typically paired with either the Boston Naming Test to form a “Language” factor (Larrabee) or are considered “Executive Functioning” tests (Strauss, Lezak, Gross, Hoogland). In the CHC model as formulated by Jewsbury, Bowden, and Duff (2016), a separate factor is estimated for these fluency tests (Schneider & McGrew, 2018; Jewsbury & Bowden, 2016). Second, the Boston Naming Test is typically either a constituent of a “Verbal” factor (Larrabee, Hoogland) or is considered as separate from the other tests considered here (Strauss, Lezak, Gross). In the CHC model, the Boston Naming Test is paired with the Story Recall variables to form the “Acquired knowledge or crystallized ability” factor. Third, the Digit Span variables are typically paired with Coding (Strauss, Lezak, Gross, Hoogland) and Trail Making Test Part A (Lezak, Gross, Hoogland). In the best-fitting CHC model, the Digit Span variables formed a separate factor and were not paired with any of these variables. Fourth, all other models, except for Hoogland, had no cross-loadings, that is, all variables only belonged to one domain. The best-fitting CHC model had three cross-loadings, with the Trail Making Test Part B measuring both “Working memory” and “Processing speed,” and Story Recall Immediate and Delayed Recall measuring both “Acquired knowledge or crystallized ability” and “Long-term memory encoding and retrieval”.

In our analyses of the CHC model, the Boston Naming Test was the only measure that was an indicator of only “Acquired knowledge or crystallized ability”, since Story Recall also functions as an indicator for “Long-term memory encoding and retrieval”. However, the Boston Naming Test is not a pure measure of crystallized ability in Jewsbury, Bowden, and Duff (2016) their specification of the CHC model, but is also an indicator of “Visuo-spatial ability” alongside tests like the Rey Complex Figure Test and Judgement of Line Orientation. In practice, the Boston Naming Test is used specifically for identifying naming deficits in patients with aphasia or dementia (Lezak, Howieson, Bigler, & Tranel, 2012, p. 551), and is not used to characterize individual differences in crystallized ability or visuo-spatial ability in cognitively healthy participants.

One caveat concerning the goodness-of-fit of the CHC model is its number of cognitive domains relative to the number of variables that were included. If there are many latent variables, and each latent variable has only indicators from a single measurement instrument, this latent variable may not represent a cognitive domain and may simply represent the variance that is unique to that particular method. In each of the models examined in this article, two test variables that belong to the same test were always assumed to measure the same latent variable. Therefore, this issue, sometimes called method variance, is not unique to the CHC model. In fact, no factor in the CHC model has only indicators belonging to a single instrument. Therefore, of the models specified here, in combination with this selection of variables, the CHC results should be relatively less determined by method variance. However, more data from a broader selection of tests is imperative.

The present article lends support to the recent endorsement of the CHC model by Jewsbury, Bowden, and Duff (2016). Their study, in which separate analyses were run for every dataset, provided a better coverage of spatial ability and fluid reasoning domains. However, the current study adds to the Jewsbury et al. findings in several ways. First, in two studies we were able to perform a single analysis of multiple datasets, thereby yielding a very large sample size. Second, the fit of the CHC model was good even though we corrected for age, sex, and level of education, which could have distorted earlier analyses. Third, we compared the CHC model to various alternatives, and even among those alternatives, the CHC model provided the best fit. Therefore, this article provides strong evidence for the CHC model.

The fact that the CHC model fits better than other models has a number of consequences for neuropsychology. First, a consequence of the cross-loadings in the CHC model is that it corroborates the view that tests generally measure more than one domain. For test selection, this does not mean that these are bad tests to administer, but rather that they can be informative for multiple domains at once. For example, if a low score on Trail Making Test Part B is observed, this could indicate impairment of “Processing speed” if observed with a low score on Trail Making Test Part A, or indicate impairment of “Working memory” if observed with a low score on Digit Span.

Second, the result has implications for the distinction between single-domain and multi-domain disorders. These disorders have typically been defined referring to the domains based on expert opinion, that is, “Executive Functions”, “Memory”, “Attention”, and so on (Petersen, 2004). Given the results, it seems better to work instead with “Long-term memory encoding and retrieval”, “Acquired knowledge or crystallized ability”, “Processing speed”, “Working memory”, and “Word fluency”. Application of the single-domain and multi-domain criteria with these domains would be straightforward. However, it is not clear whether the results that have been obtained in studies using the traditional domain definition (e.g., Ganguli et al., 2010; Libon et al., 2010) also hold with the CHC domain definition. It could be worthwhile to go back to already published data and apply the criteria using the CHC domains to study their prognostic value in comparison to that of the criteria using the traditional domains. One important domain in terms of diagnosis in the traditional model is the “Memory” domain, which is used to define amnestic variants of disorders (Tabert et al., 2006). For the CHC model, the “Long-term memory encoding and retrieval” domain could be used for the same purpose since all the same tests that load on the “Memory” factor load also on this factor.

Third, by calculating composite scores for a particular cognitive domain, one assumes that differences between people in their test scores are due to differences in their latent ability on this cognitive domain, that is, that the cognitive domain is unidimensional (Borsboom, 2008). This is done for example in the calculation of an “Executive functioning” composite score (e.g., Gross et al., 2015), where one implicitly assumes that individual variation on Trail Making Test Part B, Coding, and Digit Span Backwards is due to individual variation in Executive functioning. From a practical standpoint, calculating a composite score may be a useful form of data reduction, and the resulting latent variable may correspond well with what is known from the literature (Preacher & MacCallum, 2003). Therefore, there is something to be said for calculating such a composite score, from a constructivist perspective (Borsboom, 2008). From a statistical perspective though, an “Executive functioning” composite score does not seem to represent a unitary construct. The variables that are typically assigned to the “Executive Functioning” domain are spread out over three domains in the best-fitting CHC model (“Processing speed”, “Working memory”, and “Word fluency”), suggesting that unidimensionality is violated. Fourth, it should be recognized that in both analyses, all latent variables were correlated in the CHC model. The influence of age and level of education that could have artificially produced such a correlation were partialed out. Therefore, although the tests in neuropsychological practice are designed to measure well-separable cognitive domains, these domains do not in fact seem completely separable. This could be due to the design of the tests. Perhaps tests have not been designed such that they can specifically measure individual variation only in “Working memory” while not also measuring variation in “Processing speed.” However, this could also be due to the nature of cognitive functioning. All cognitive functions could be so deeply intertwined that it is not possible to measure one without the other (Van der Maas et al., 2006).

Although in this article there was an emphasis on the differences between factor models, we can as easily focus on the communalities. As one reviewer pointed out, the Lezak model is highly similar to all other models. This is not surprising, as the test variables included here were once devised with certain measurement goals in mind. For example, Story Recall and the Verbal Learning Test were devised for the measurement of memory and were subsumed under the same latent variable in all models considered here. Therefore, the differences between models necessarily focus on the variables like the Boston Naming Test and Fluency, which are less easily categorized. None of the models that were derived from the literature was proven wrong, although the one factor model did fit considerably worse than the other models.

It is important to realize the limitations of our results. First, the goal was to establish a factor model for cognitively healthy participants although some participants included in the analyses may not have been cognitively healthy. Some of the contributing studies did not have the explicit goal to exclude pathology, but instead had the goal to obtain a representative sample from the population. This is true for both studies 1 and 2.

Second, we should be careful not to overgeneralize the results to other samples. Tests loading on the same latent variable are not necessarily redundant measures of the same latent variable in all samples. For example, immediate recall and delayed recall on the Verbal Learning Tests were found to be indicators of the same latent variable in the CHC model. However, it has been argued immediate and delayed recall are not interchangeable tests in clinical practice since the function of one may be disrupted by disorder or injury while the other remains intact (Delis, Jacobson, Bondi, Hamilton, & Salmon, 2003). Many neuropsychological tasks show particular sensitivities to specific disorders. For example, aphasia may have specific effects on language-based tasks that have little to do with the higher-order factor structure proposed here. If neuropsychological tests would measure different latent variables in different populations, this would invalidate standard neuropsychological assessment practice. However, investigations into measurement invariance, which statistically evaluate whether the same factor structure holds for different populations (Widaman & Reise, 1997), have shown that the same factor structure holds for many populations in neuropsychology (Bowden, Cook, Bardenhagen, Shores, & Carstairs, 2004; Park et al., 2012; Schretlen et al., 2013). Therefore, measurement invariance seems to be the rule rather than the exception.

Third, in working with correlations between variables, the underlying assumption is that the relations between variables can be captured by a linear model. This assumption may not be tenable for every pair of variables. Especially for the relation between age and some test variables, the relation may be better characterized by a curve, rather than a straight line, with an increased deterioration for the very old (Salthouse, 2009). Non-linearity may affect partial correlations in a variety of ways such as masking partial correlations or introducing spurious partial correlations (Vargha, Bergman, & Delaney, 2013). However, although non-linear effects are found, effects are still largely linear for most ages (rather than for example an inverted U-shape). Therefore, the effect of the violation of the linearity assumption would be a small undercorrection for the effect of age for the very old.

Fourth, only parts of the models that were described in the introduction were tested in this study. For example, far from all variables that are mentioned in Lezak, Howieson, Bigler, and Tranel (2012) could be included, and not all latent variables that are included in their overview could be included in the latent structure of the model. This was the case for all models. After all, just twelve test variables were included in study 1 and ten variables were included in study 2, whereas many more test variables are used in clinical neuropsychology. However, these ten to twelve variables are not a random selection from the field, as these variables are the most commonly used and form the basis of many neuropsychological assessments.

Fifth, there was a high correlation between two latent variables of the CHC model, “long-term memory encoding and retrieval” and “acquired knowledge or crystallized ability.” Jewsbury, Bowden, and Duff (2016) ran into similar issues, for some datasets, wherein factors were so highly correlated that there were problems in fitting the model (described in their supplemental material). Although the correlation between the latent variables in Study 1 is statistically different from 1 (95% CI: 0.95–0.99), we do not consider these latent variables to be distinguishable with the current selection of variables. Therefore, it was considered to collapse these two latent variables into one. However, such exploratory adaptations to the model could be overfitting this particular selection of variables and would not fit our confirmatory setup. As Jewsbury, Bowden, and Duff (2016) show, these two factors are meaningful when considering other selections of tests.

To our knowledge, MASEM has not been utilized in a large scale project like this but has been limited to the structure of small sets of variables. One other source of large scale data that would be amenable to this type of study, specifically to study the CHC model, would be the datasets that Carroll used to study the structure of intelligence, collected in the Human Cognitive Abilities project (McGrew, 2009). Furthermore, with correlation matrices from newly published studies, the present meta-analysis could be extended to include other variables and to include variance parameters on the correlations. To facilitate such an analysis, we provide correlation matrices in the appendix. We recommend that, as a rule, correlation matrices are shared publicly in articles or in supplemental materials, to facilitate the type of meta-analysis presented here.

To conclude, in two independent large-scale analyses the Cattell-Horn-Carroll (CHC) model best describes the structure of neuropsychological test domains.

### Electronic supplementary material

ESM 1 (DOCX 15 kb)

ESM 2 (PDF 399 kb)

## Figures and Tables

**Fig. 4:**
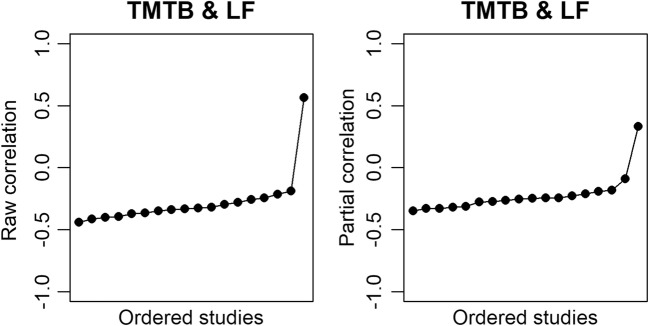
Bivariate raw and partial correlations between Trail Making Test B and Letter Fluency, plotted for different studies. The studies are ordered by the size of the correlation

**Fig. 5:**
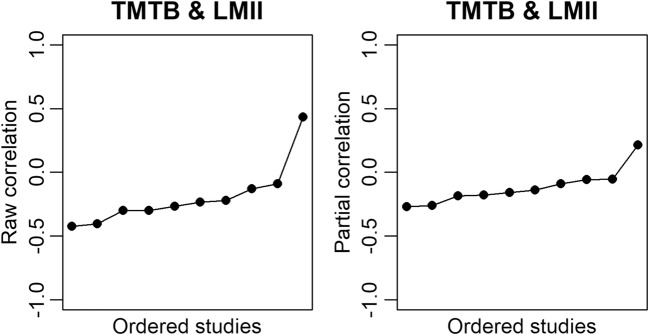
Bivariate raw and partial correlations between Trail Making Test B and Story Recall Delayed Recall, plotted for different studies. The studies are ordered by the size of the correlation

## Appendix 1: Search terms used in PsycINFO

### #1 Factor model

factor analysis/ OR factor structure/ OR structural equation modeling/ OR (factor* model* OR factor* analy* OR structural equation* model* OR EFA OR CFA OR SEM OR factor* structur* OR confirmatory factor* OR exploratory factor*).ti,ab,id.

### #2 Specific neuropsychological tests

stroop color word test/ OR stroop effect/ OR wechsler memory scale/ OR wisconsin card sorting test/ OR verbal learn*.tm. OR ((clock* AND (test* OR draw*)) OR (tower AND (test* OR london OR hanoi)) OR benton OR vis* retent* OR BVRT OR fac* recogni* OR BFRT OR judg* of line* OR line orientation OR JLO OR BJLO OR JOLO OR block design OR blockdesign OR Kohs OR boston naming OR BNT OR brixton OR spatial anticipation OR BSAT OR card sort* task* OR card sort* test* OR cardsort* test* OR WCST OR MWCST OR complex figur* OR rcf* OR rocf* OR rey-osterrieth OR digit* span* OR digitspan OR (span* ADJ1 (forward* OR back*)) OR spanforward OR digit* symbol* OR symbol* substitution* OR symbol coding OR DSST* OR family pictures OR figur* fluency OR groov* peg* OR purdue peg* OR pegboard OR letter fluency OR cowat OR controlled oral word association OR controlled word association OR controlled association* OR letter number OR LNS OR location learning OR LLT OR logical memory OR matr* reas* OR object* assemb* OR pac* audit* seri* additi* OR PASAT OR pict* arrangement* OR pict* compl* OR rivermead behavio* OR rbmt* OR selecti* remindi* OR srt OR Buschke OR VSRT OR semantic fluency OR verbal fluency OR category fluency OR animal* naming OR occupation* naming OR spatial span OR stroop OR symbol* search* OR trail making OR trial making OR tmt OR halstead reitan OR verbal learn* test* OR verbal learn* task* OR RAVLT* OR AVLT* OR CVLT* OR HVLT* OR verbal pair* associat* OR visual reproduction OR WMS*).ti,ab,id,tm.

### #3 Clinical neuropsychological test batteries

(test battery/ OR (((tests OR test scores OR test results) ADJ2 (attention* OR cognit* OR memory OR neuropsych* OR visual OR visuospatial* OR visuomotor OR verbal* OR executive OR learning OR IQ OR motor OR auditory OR perception OR inhibit* OR psychometr*)) OR (test* AND battery)).ti,ab,id,tm.) AND (neuropsychol*).ti,ab,id,hw,jx.

**1 AND (2 OR 3).**

## Appendix 2: Test variables of interest

Trail Making Test A, Trail Making Test B, Stroop Color, Stroop Word, Stroop Color-Word, Letter Fluency / FAS / COWAT, Semantic Fluency / Category Fluency / Animal Naming, Verbal Learning Test Total, Verbal Learning Test Recall, Verbal Learning Test Recognition, WAIS Vocabulary, WAIS Similarities, WAIS Information, WAIS Arithmetic, WAIS Letter Number Sequencing, WAIS Comprehension, WAIS Picture Completion, WAIS Block Design, WAIS Matrix Reasoning, WAIS Digit Symbol Substitution / Coding, WAIS Symbol Search, WAIS Picture Arrangement, WAIS Object Assembly, Logical Memory / Story Immediate, Logical Memory / Story Delayed, WMS Faces Immediate, WMS Faces Delayed, WMS Verbal Paired Associates Immediate, WMS Verbal Paired Associates Delayed, WMS Visual Paired Associates, WMS Family Pictures Immediate, WMS Family Pictures Delayed, WMS Visual Reproduction, WMS Spatial Span, Digit Span Forward, Digit Span Backward, Rey Complex Figure Copy, Rey Complex Figure Immediate Recall, Rey Complex Figure Delayed Recall, Raven Progressive Matrices, Wisconsin Number of Categories, Wisconsin Number of Perseverative Errors, Wisconsin Number of Perseverative Responses, Token Test Score, Grooved Pegboard Dominant, Grooved Pegboard Non-dominant, Benton Visual Retention Test, Brixton Spatial Anticipation, Rivermead Immediate 1 + 2, Rivermead Delayed 1 + 2, Clock Drawing Test, Boston Naming Test, Ruff Figural Fluency Test, Ruff 2 and 7, Buschke Selective Reminding Test Total Recall (TR), Buschke Selective Reminding Test Long Term Retrieval (LTR), Buschke Selective Reminding Test Long Term Storage (LTS), Buschke Selective Reminding Test Consistent Long Term Retrieval (CLTR), Free and Cued Selective Reminding Test (FCSRT), Buschke Selective Reminding Test Delayed Recall (DR), Benton Facial Recognition Test, Symbol Digit Modalities Test, Brief Visuospatial Memory Test, Judgement of Line Orientation, Tower of London Total number of moves, Continuous Performance Test (d’), Peabody Picture Vocabulary Test, PASAT Total number correct, BADS Zoo map, BADS Key search.

## Appendix 3: Analysis without TMT B from Royall, Bishnoi, and Palmer (2015)

Figure 4, left hand panel, shows that one correlation between Trail Making Test B and Letter Fluency is exceptional, in that is positive and large. This is also the case for the correlation between Trail Making Test B and Story Recall Delayed Recall from this study in Fig. 5, so it is not Letter Fluency that is at fault. These findings remain after partialing out the effect of age, sex, and level of education (right hand panel). This could be a case of a coding error, but Royall, Bishnoi, and Palmer (2015) is clear that the Trail Making Test B variable refers to the score in seconds, like other studies. Royall, Bishnoi, and Palmer (2015) also note that the correlations with Trail Making Test B seem strange. One last option is that it is simply due to sampling variance. However, given that this concerns an impressive 875 participants, this is unlikely. Other correlations that seemed different from the rest came from much smaller studies.

All correlations with Trail Making Test B were removed from the Royall correlation matrix for the main analysis, leaving Story Recall Delayed Recall, Boston Naming Test and Letter Fluency. We did however run the analysis with these correlations with Trail Making Test B included. The results are given in Table 7. The conclusions do not differ from the conclusions of the main analysis: The second CHC model was considered best in this analysis as well.

**Table 7:** Comparison results with correlations with TMT B from Royall, Bishnoi, and Palmer (2015) included

	RMSEA	SRMR	CFI	AIC	BIC
Gross et al. (2015)*	–	–	–	–	–
Hoogland et al. (2017)*	–	–	–	–	–
CHC 1* (Schneider & McGrew, 2018)	–	–	–	–	–
One factor (Verhaeghen & Salthouse, 1997)	0.058	0.218	0.937	11,085.5	10,599
Lezak, Howieson, Bigler, and Tranel (2012)	0.041	0.122	0.973	4790.3	4357.9
Strauss, Sherman, and Spreen (2006)	0.038	0.112	0.979	3740.7	3344.4
Larrabee (2014)	0.032	0.099	0.983	2913.2	2480.7
CHC 2 (Schneider & McGrew, 2018)	0.023	0.059	0.993	1225.2	855.9

*Model did not converge

## Appendix 4: Study characteristics and correlation matrices

**Table:**

Adrover-Roig, D., Sesé, A., Barceló, F., & Palmer, A. (2012). A latent variable approach to executive control in healthy ageing. *Brain and Cognition*, *78*(3), 284–299. doi:10.1016/j.bandc.2012.01.005	
*N* = 122	
Sex coding: male > female	
Education coding: higher is better	
Correlation matrix available from original publication

**Table:**

	AGE	SEX	EDU	TMTA	TMTB	SR-IR	SR-DR	SF	BNT	VLT-TR	VLT-DR
AGE	1	−0.121	−0.072	0.193	0.143	−0.241	−0.22	−0.156	−0.071	−0.128	−0.029
SEX	−0.121	1	−0.23	0.116	0.009	0.19	0.178	−0.036	−0.021	−0.357	−0.277
EDU	−0.072	−0.23	1	−0.198	−0.276	0.066	0.028	−0.104	0.201	0.08	0.042
TMTA	0.193	0.116	−0.198	1	0.403	0.012	−0.021	0.243	−0.011	−0.052	−0.183
TMTB	0.143	0.009	−0.276	0.403	1	−0.016	−0.091	0.259	0.092	0.2	0.063
SR-IR	−0.241	0.19	0.066	0.012	−0.016	1	0.864	0.083	0.274	0.069	0.043
SR-DR	−0.22	0.178	0.028	−0.021	−0.091	0.864	1	0.131	0.215	0.048	0.036
SF	−0.156	−0.036	−0.104	0.243	0.259	0.083	0.131	1	0.119	0.333	0.223
BNT	−0.071	−0.021	0.201	−0.011	0.092	0.274	0.215	0.119	1	0.145	0.061
VLT-TR	−0.128	−0.357	0.08	−0.052	0.2	0.069	0.048	0.333	0.145	1	0.567
VLT-DR	−0.029	−0.277	0.042	−0.183	0.063	0.043	0.036	0.223	0.061	0.567	1

Andrejeva, N., Knebel, M., Dos Santos, V., Schmidt, J., Herold, C. J., Tudoran, R., ... & Gorenc-Mahmutaj, L. (2016). Neurocognitive deficits and effects of cognitive reserve in mild cognitive impairment. *Dementia and Geriatric Cognitive Disorders*, *41*(3–4), 199–209. doi:10.1159/000443791

*N* = 65

Sex coding: female > male

Education coding: higher is better

**Table:**

Andreotti, C., & Hawkins, K. A. (2015). RBANS norms based on the relationship of age, gender, education, and WRAT-3 reading to performance within an older African American sample. *The Clinical Neuropsychologist*, *29*(4), 442–465. doi:10.1080/13854046.2015.1039589	
*N* = 289	
Sex coding: Sex not included	
Education coding: higher is better	
Correlation matrix available from original publication

**Table:**

	AGE	SEX	EDU	TMTA	TMTB	SR-IR	SR-DR
AGE	1	−0.011	−0.218	0.311	0.398	−0.21	−0.227
SEX	−0.011	1	−0.096	−0.075	−0.038	0.11	0.12
EDU	−0.218	−0.096	1	−0.155	−0.286	0.311	0.307
TMTA	0.311	−0.075	−0.155	1	0.57	−0.206	−0.211
TMTB	0.398	−0.038	−0.286	0.57	1	−0.281	−0.299
SR-IR	−0.21	0.11	0.311	−0.206	−0.281	1	0.86
SR-DR	−0.227	0.12	0.307	−0.211	−0.299	0.86	1

Albert, M., Massaro, J., DeCarli, C., Beiser, A., Seshadri, S., Wolf, P. A., & Au, R. (2010). Profiles by sex of brain MRI and cognitive function in the framingham offspring study. *Alzheimer Disease and Associated Disorders*, *24*(2), 190–193. doi:10.1097/WAD.0b013e3181c1ed44

*N* = 2085

Sex coding: female > male

Education coding: higher is better

**Table:**

	AGE	SEX	EDU	SR-IR	SR-DR	SF	DSF	DSB	COD	BNT	VLT-TR	VLT-DR
AGE	1	−0.013	−0.186	−0.258	−0.279	−0.321	−0.121	−0.097	−0.382	−0.185	−0.352	−0.339
SEX	−0.013	1	0.133	−0.064	−0.081	−0.115	0.042	−0.022	−0.077	0.065	−0.133	−0.126
EDU	−0.186	0.133	1	0.269	0.252	0.245	0.152	0.212	0.293	0.168	0.225	0.203
SR-IR	−0.258	−0.064	0.269	1	0.864	0.387	0.179	0.277	0.358	0.272	0.454	0.469
SR-DR	−0.279	−0.081	0.252	0.864	1	0.419	0.173	0.273	0.381	0.294	0.489	0.54
SF	−0.321	−0.115	0.245	0.387	0.419	1	0.203	0.29	0.498	0.337	0.491	0.475
DSF	−0.121	0.042	0.152	0.179	0.173	0.203	1	0.465	0.21	0.15	0.233	0.157
DSB	−0.097	−0.022	0.212	0.277	0.273	0.29	0.465	1	0.326	0.17	0.32	0.232
COD	−0.382	−0.077	0.293	0.358	0.381	0.498	0.21	0.326	1	0.381	0.424	0.407
BNT	−0.185	0.065	0.168	0.272	0.294	0.337	0.15	0.17	0.381	1	0.255	0.267
VLT-TR	−0.352	−0.133	0.225	0.454	0.489	0.491	0.233	0.32	0.424	0.255	1	0.727
VLT-DR	−0.339	−0.126	0.203	0.469	0.54	0.475	0.157	0.232	0.407	0.267	0.727	1

Barnes, L. L., Yumoto, F., Capuano, A., Wilson, R. S., Bennett, D. A., & Tractenberg, R. E. (2016). Examination of the factor structure of a global cognitive function battery across race and time. *Journal of the International Neuropsychological Society*, *22*(1), 66–75. doi:10.1017/S1355617715001113

*N* = 2854

Sex coding: male > female

Education coding: higher is better

**Table:**

	AGE	SEX	EDU	TMTA	TMTB	SR-IR	SR-DR	LF	SF	DSF	DSB	VLT-TR	VLT-DR
AGE	1	0.027	0.263	0.48	0.496	−0.292	−0.39	−0.002	−0.248	−0.235	−0.043	−0.32	−0.305
SEX	0.027	1	0.157	0.02	0.066	0.015	−0.029	0.106	0.19	0.153	0.221	−0.15	−0.155
EDU	0.263	0.157	1	0.1	0.002	0.044	0.086	0.085	0.082	−0.093	0.046	0.03	0.057
TMTA	0.48	0.02	0.1	1	0.718	−0.144	−0.211	−0.187	−0.375	−0.116	−0.136	−0.187	−0.143
TMTB	0.496	0.066	0.002	0.718	1	−0.34	−0.424	−0.215	−0.295	−0.268	−0.395	−0.338	−0.257
SR-IR	−0.292	0.015	0.044	−0.144	−0.34	1	0.877	0.084	0.325	0.253	0.34	0.467	0.523
SR-DR	−0.39	−0.029	0.086	−0.211	−0.424	0.877	1	0.166	0.293	0.235	0.339	0.51	0.621
LF	−0.002	0.106	0.085	−0.187	−0.215	0.084	0.166	1	0.223	0.252	0.319	0.173	0.097
SF	−0.248	0.19	0.082	−0.375	−0.295	0.325	0.293	0.223	1	0.156	0.22	0.2	0.146
DSF	−0.235	0.153	−0.093	−0.116	−0.268	0.253	0.235	0.252	0.156	1	0.384	0.319	0.196
DSB	−0.043	0.221	0.046	−0.136	−0.395	0.34	0.339	0.319	0.22	0.384	1	0.284	0.232
VLT-TR	−0.32	−0.15	0.03	−0.187	−0.338	0.467	0.51	0.173	0.2	0.319	0.284	1	0.769
VLT-DR	−0.305	−0.155	0.057	−0.143	−0.257	0.523	0.621	0.097	0.146	0.196	0.232	0.769	1

Bennett, I. J., & Stark, C. E. (2016). Mnemonic discrimination relates to perforant path integrity: an ultra-high resolution diffusion tensor imaging study. *Neurobiology of Learning and Memory*, *129*, 107–112. doi:10.1016/j.nlm.2015.06.014

*N* = 109

Sex coding: male > female

Education coding: higher is better

**Table:**

	AGE	SEX	EDU	TMTB	SR-IR	SR-DR	LF	SF	DSF	DSB	BNT
AGE	1	0.012	−0.154	0.39	−0.177	−0.228	−0.171	−0.359	−0.213	−0.225	−0.264
SEX	0.012	1	−0.164	0.007	−0.121	−0.108	0.044	0.063	0.022	−0.087	−0.234
EDU	−0.154	−0.164	1	−0.296	0.244	0.273	0.246	0.357	0.304	0.277	0.275
TMTB	0.39	0.007	−0.296	1	−0.157	−0.22	−0.331	−0.444	−0.257	−0.271	−0.335
SR-IR	−0.177	−0.121	0.244	−0.157	1	0.87	0.262	0.224	0.153	0.301	0.419
SR-DR	−0.228	−0.108	0.273	−0.22	0.87	1	0.287	0.308	0.189	0.284	0.432
LF	−0.171	0.044	0.246	−0.331	0.262	0.287	1	0.541	0.3	0.349	0.331
SF	−0.359	0.063	0.357	−0.444	0.224	0.308	0.541	1	0.305	0.322	0.368
DSF	−0.213	0.022	0.304	−0.257	0.153	0.189	0.3	0.305	1	0.493	0.168
DSB	−0.225	−0.087	0.277	−0.271	0.301	0.284	0.349	0.322	0.493	1	0.3
BNT	−0.264	−0.234	0.275	−0.335	0.419	0.432	0.331	0.368	0.168	0.3	1

Bezdicek, O., Libon, D. J., Stepankova, H., Panenkova, E., Lukavsky, J., Garrett, K. D., ... & Kopecek, M. (2014). Development, validity, and normative data study for the 12-word Philadelphia Verbal Learning Test [czP (r) VLT-12] among older and very old Czech adults. *The Clinical Neuropsychologist*, *28*(7), 1162–1181. doi:10.1080/13854046.2014.952666

*N* = 540

Sex coding: female > male

Education coding: higher is better

**Table:**

	AGE	SEX	EDU	SR-IR	SR-DR	SF	DSB	COD
AGE	1	0.02	−0.071	−0.18	−0.169	−0.144	−0.141	−0.198
SEX	0.02	1	−0.029	0.077	0.108	0.059	−0.041	0.162
EDU	−0.071	−0.029	1	0.307	0.287	0.241	0.199	0.285
SR-IR	−0.18	0.077	0.307	1	0.873	0.197	0.238	0.238
SR-DR	−0.169	0.108	0.287	0.873	1	0.195	0.23	0.244
SF	−0.144	0.059	0.241	0.197	0.195	1	0.28	0.342
DSB	−0.141	−0.041	0.199	0.238	0.23	0.28	1	0.264
COD	−0.198	0.162	0.285	0.238	0.244	0.342	0.264	1

Booth, T., Royle, N. A., Corley, J., Gow, A. J., Hernández, M. D. C. V., Maniega, S. M., ... & Deary, I. J. (2015). Association of allostatic load with brain structure and cognitive ability in later life. *Neurobiology of Aging*, *36*(3), 1390–1399. doi:10.1016/j.neurobiolaging.2014.12.020

*N* = 970

Sex coding: female > male

Education coding: higher is better

**Table:**

	AGE	SEX	EDU	LF	SF
AGE	1	0.093	−0.229	−0.324	−0.23
SEX	0.093	1	−0.099	−0.189	−0.067
EDU	−0.229	−0.099	1	0.312	0.038
LF	−0.324	−0.189	0.312	1	0.411
SF	−0.23	−0.067	0.038	0.411	1

Bouazzaoui, B., Fay, S., Taconnat, L., Angel, L., Vanneste, S., & Isingrini, M. (2013). Differential involvement of knowledge representation and executive control in episodic memory performance in young and older adults. *Canadian Journal of Experimental Psychology/Revue Canadienne de Psychologie Expérimentale*, *67*(2), 100–107. doi:10.1037/a0028517

*N* = 120

Sex coding: female > male

Education coding: higher is better

**Table:**

	AGE	SEX	EDU	SR-IR	SR-DR	DSF	DSB	COD	VLT-TR	VLT-DR
AGE	1	0.017	0.018	0.002	0.009	−0.082	−0.025	−0.148	−0.025	−0.045
SEX	0.017	1	−0.028	0.16	0.164	−0.076	−0.01	0.315	0.248	0.226
EDU	0.018	−0.028	1	0.189	0.188	0.132	0.182	0.238	0.278	0.176
SR-IR	0.002	0.16	0.189	1	0.916	0.133	0.21	0.252	0.533	0.468
SR-DR	0.009	0.164	0.188	0.916	1	0.121	0.196	0.238	0.531	0.51
DSF	−0.082	−0.076	0.132	0.133	0.121	1	0.57	0.167	0.128	0.035
DSB	−0.025	−0.01	0.182	0.21	0.196	0.57	1	0.247	0.289	0.173
COD	−0.148	0.315	0.238	0.252	0.238	0.167	0.247	1	0.332	0.296
VLT-TR	−0.025	0.248	0.278	0.533	0.531	0.128	0.289	0.332	1	0.745
VLT-DR	−0.045	0.226	0.176	0.468	0.51	0.035	0.173	0.296	0.745	1

Bowden, S. C., Cook, M. J., Bardenhagen, F. J., Shores, E. A., & Carstairs, J. R. (2004). Measurement invariance of core cognitive abilities in heterogeneous neurological and community samples. *Intelligence*, *32*(4), 363–389. doi:10.1016/j.intell.2004.05.002

*N* = 399

Sex coding: female > male

Education coding: higher is better

**Table:**

	AGE	SEX	EDU	SF	COD
AGE	1	−0.07	−0.07	−0.24	−0.33
SEX	−0.07	1	0.17	0.07	0.02
EDU	−0.07	0.17	1	0.16	0.34
SF	−0.24	0.07	0.16	1	0.47
COD	−0.33	0.02	0.34	0.47	1

Bunce, D., Batterham, P. J., Christensen, H., & Mackinnon, A. J. (2014). Causal associations between depression symptoms and cognition in a community-based cohort of older adults. *The American Journal of Geriatric Psychiatry*, *22*(12), 1583–1591. doi:10.1016/j.jagp.2014.01.004

*N* = 853

Sex coding: male > female

Education coding: higher is better

**Table:**

Chan, R. C., Wang, Y., Wang, L., Chen, E. Y., Manschreck, T. C., Li, Z. J., ... & Gong, Q. Y. (2009). Neurological soft signs and their relationships to neurocognitive functions: A re-visit with the structural equation modeling design. *PLoS One*, *4*(12), 1–8. doi:10.1371/journal.pone.0008469	
*N* = 160	
Sex coding: male > female	
Education coding: higher is better

**Table:**

	AGE	SEX	EDU	TMTA	TMTB	SR-IR	SR-DR	SF	DSB
AGE	1	0.238	−0.089	0.376	0.424	−0.336	−0.301	−0.349	−0.257
SEX	0.238	1	0.287	−0.038	0.007	−0.027	−0.023	−0.36	−0.036
EDU	−0.089	0.287	1	−0.373	−0.25	0.302	0.324	−0.017	0.289
TMTA	0.376	−0.038	−0.373	1	0.529	−0.297	−0.302	−0.292	−0.311
TMTB	0.424	0.007	−0.25	0.529	1	−0.348	−0.3	−0.257	−0.259
SR-IR	−0.336	−0.027	0.302	−0.297	−0.348	1	0.89	0.358	0.372
SR-DR	−0.301	−0.023	0.324	−0.302	−0.3	0.89	1	0.332	0.362
SF	−0.349	−0.36	−0.017	−0.292	−0.257	0.358	0.332	1	0.238
DSB	−0.257	−0.036	0.289	−0.311	−0.259	0.372	0.362	0.238	1

Chen, Y. C., Jung, C. C., Chen, J. H., Chiou, J. M., Chen, T. F., Chen, Y. F., ... & Lee, M. S. (2017). Association of dietary patterns with global and domain-specific cognitive decline in Chinese elderly. *Journal of the American Geriatrics Society*, *65*(6), 1159–1167. doi:10.1111/jgs.14741

*N* = 475

Sex coding: male > female

Education coding: higher is better

**Table:**

	AGE	SEX	EDU	TMTB	LF	DSF	DSB	COD	VLT-TR	VLT-DR
AGE	1	−0.112	−0.305	0.664	−0.314	−0.4	−0.657	−0.458	−0.532	−0.441
SEX	−0.112	1	0.238	0.064	−0.02	0.169	0.172	0.38	−0.201	−0.218
EDU	−0.305	0.238	1	−0.339	0.368	0.531	0.466	0.191	0.138	0.259
TMTB	0.664	0.064	−0.339	1	−0.415	−0.299	−0.555	−0.641	−0.404	−0.47
LF	−0.314	−0.02	0.368	−0.415	1	0.326	0.442	0.102	0.444	0.536
DSF	−0.4	0.169	0.531	−0.299	0.326	1	0.563	0.299	0.238	0.185
DSB	−0.657	0.172	0.466	−0.555	0.442	0.563	1	0.349	0.454	0.384
COD	−0.458	0.38	0.191	−0.641	0.102	0.299	0.349	1	0.184	0.244
VLT-TR	−0.532	−0.201	0.138	−0.404	0.444	0.238	0.454	0.184	1	0.785
VLT-DR	−0.441	−0.218	0.259	−0.47	0.536	0.185	0.384	0.244	0.785	1

Ciccarelli, N., Fabbiani, M., Baldonero, E., Fanti, I., Cauda, R., Giambenedetto, S. D., & Silveri, M. C. (2012). Effect of aging and human immunodeficiency virus infection on cognitive abilities. *Journal of the American Geriatrics Society*, *60*(11), 2048–2055. doi:10.1111/j.1532-5415.2012.04213.x

N = 39

Sex coding: male > female

Education coding: higher is better

**Table:**

	AGE	SEX	EDU	TMTA	TMTB	SR-IR	SR-DR	DSF	DSB	VLT-TR	VLT-DR
AGE	1	−0.042	0.055	0.312	0.312	−0.09	−0.11	−0.048	−0.023	−0.225	−0.161
SEX	−0.042	1	−0.073	−0.043	−0.024	−0.029	−0.026	−0.046	−0.017	0.157	0.147
EDU	0.055	−0.073	1	0.019	−0.12	0.238	0.237	0.166	0.215	0.136	0.148
TMTA	0.312	−0.043	0.019	1	0.496	−0.091	−0.11	−0.107	−0.13	−0.239	−0.18
TMTB	0.312	−0.024	−0.12	0.496	1	−0.222	−0.234	−0.219	−0.255	−0.275	−0.214
SR-IR	−0.09	−0.029	0.238	−0.091	−0.222	1	0.897	0.166	0.266	0.442	0.407
SR-DR	−0.11	−0.026	0.237	−0.11	−0.234	0.897	1	0.13	0.232	0.461	0.467
DSF	−0.048	−0.046	0.166	−0.107	−0.219	0.166	0.13	1	0.562	0.17	0.056
DSB	−0.023	−0.017	0.215	−0.13	−0.255	0.266	0.232	0.562	1	0.24	0.16
VLT-TR	−0.225	0.157	0.136	−0.239	−0.275	0.442	0.461	0.17	0.24	1	0.766
VLT-DR	−0.161	0.147	0.148	−0.18	−0.214	0.407	0.467	0.056	0.16	0.766	1

Darst, B. F., Koscik, R. L., Hermann, B. P., La Rue, A., Sager, M. A., Johnson, S. C., & Engelman, C. D. (2015). Heritability of cognitive traits among siblings with a parental history of Alzheimer’s disease. *Journal of Alzheimer’s Disease*, *45*(4), 1149–1155. doi:10.3233/JAD-142658

*N* = 1226

Sex coding: female > male

Education coding: higher is better

**Table:**

	AGE	SEX	EDU	SR-IR	SR-DR	SF	DSF	COD	BNT	VLT-TR	VLT-DR
AGE	1	−0.062	−0.007	−0.205	−0.247	−0.191	−0.059	−0.424	−0.155	−0.249	−0.23
SEX	−0.062	1	0.18	0.016	−0.053	−0.178	0.09	−0.07	0.183	−0.1	−0.171
EDU	−0.007	0.18	1	0.248	0.209	0.085	0.187	0.221	0.275	0.192	0.108
SR-IR	−0.205	0.016	0.248	1	0.787	0.343	0.291	0.383	0.313	0.582	0.55
SR-DR	−0.247	−0.053	0.209	0.787	1	0.344	0.206	0.424	0.318	0.575	0.623
SF	−0.191	−0.178	0.085	0.343	0.344	1	0.168	0.416	0.225	0.393	0.371
DSF	−0.059	0.09	0.187	0.291	0.206	0.168	1	0.213	0.133	0.275	0.113
COD	−0.424	−0.07	0.221	0.383	0.424	0.416	0.213	1	0.356	0.433	0.377
BNT	−0.155	0.183	0.275	0.313	0.318	0.225	0.133	0.356	1	0.243	0.232
VLT-TR	−0.249	−0.1	0.192	0.582	0.575	0.393	0.275	0.433	0.243	1	0.65
VLT-DR	−0.23	−0.171	0.108	0.55	0.623	0.371	0.113	0.377	0.232	0.65	1

Duff, K. D., Langbehn, D. R., Schoenberg, M. R., Moser, D. J., Baade, L. E., Mold, J. W.,. .. Adams, R. L. (2006). Examining the repeatable battery for the assessment of neuropsychological status: Factor analytic studies in an elderly sample. *The American Journal of Geriatric Psychiatry, 14*, 976–979. doi:10.1097/01.JGP.0000229690.70011

*N* = 823

Sex coding: male > female

Education coding: higher is better

**Table:**

	AGE	SEX	EDU	TMTA	TMTB	SF	COD	VLT-TR
AGE	1	−0.104	0.436	−0.038	−0.053	0.427	−0.118	0.099
SEX	−0.104	1	−0.025	−0.052	−0.057	0.042	0.368	0.085
EDU	0.436	−0.025	1	−0.225	−0.168	0.301	0.153	0.318
TMTA	−0.038	−0.052	−0.225	1	0.486	−0.314	−0.353	−0.043
TMTB	−0.053	−0.057	−0.168	0.486	1	−0.351	−0.269	−0.143
SF	0.427	0.042	0.301	−0.314	−0.351	1	0.093	0.171
COD	−0.118	0.368	0.153	−0.353	−0.269	0.093	1	0.325
VLT-TR	0.099	0.085	0.318	−0.043	−0.143	0.171	0.325	1

Eifler, S., Rausch, F., Schirmbeck, F., Veckenstedt, R., Englisch, S., Meyer-Lindenberg, A., ... & Zink, M. (2014). Neurocognitive capabilities modulate the integration of evidence in schizophrenia. *Psychiatry Research*, *219*(1), 72–78. doi:10.1016/j.psychres.2014.04.056

*N* = 52

Sex coding: female > male

Education coding: higher is better

**Table:**

	AGE	SEX	EDU	TMTA	SR-IR	SR-DR	DSF	DSB	VLT-TR
AGE	1	−0.011	−0.407	−0.028	−0.239	−0.443	−0.241	−0.161	−0.367
SEX	−0.011	1	−0.286	0.222	−0.111	−0.282	0.154	0.151	−0.217
EDU	−0.407	−0.286	1	−0.264	0.3	0.187	0.058	0.088	0.476
TMTA	−0.028	0.222	−0.264	1	−0.367	−0.3	−0.289	−0.253	−0.202
SR-IR	−0.239	−0.111	0.3	−0.367	1	0.339	0.06	0.067	0.387
SR-DR	−0.443	−0.282	0.187	−0.3	0.339	1	−0.03	−0.062	0.071
DSF	−0.241	0.154	0.058	−0.289	0.06	−0.03	1	0.695	0.348
DSB	−0.161	0.151	0.088	−0.253	0.067	−0.062	0.695	1	0.435
VLT-TR	−0.367	−0.217	0.476	−0.202	0.387	0.071	0.348	0.435	1

Fernaeus, S. E., Östberg, P., Wahlund, L. O., & Hellström, Å. (2014). Memory factors in Rey AVLT: implications for early staging of cognitive decline. *Scandinavian Journal of Psychology*, *55*(6), 546–553. doi:10.1111/sjop.12157

*N* = 42

Sex coding: male > female

Education coding: higher is better

**Table:**

	AGE	SEX	EDU	LF	SF	DSF	DSB
AGE	1	−0.264	0.213	−0.054	−0.066	0.058	−0.266
SEX	−0.264	1	−0.139	0.152	0.287	0.262	0.356
EDU	0.213	−0.139	1	0.243	0.28	0.177	−0.024
LF	−0.054	0.152	0.243	1	0.539	0.24	0.48
SF	−0.066	0.287	0.28	0.539	1	0.42	0.501
DSF	0.058	0.262	0.177	0.24	0.42	1	0.508
DSB	−0.266	0.356	−0.024	0.48	0.501	0.508	1

Ferreira, N. V., Cunha, P. J., da Costa, D. I., dos Santos, F., Costa, F. O., Consolim-Colombo, F., & Irigoyen, M. C. (2015). Association between functional performance and executive cognitive functions in an elderly population including patients with low ankle–brachial index. *Clinical Interventions in Aging*, *10*, 839–847. doi:10.2147/CIA.S69270

*N* = 40

Sex coding: female > male

Education coding: higher is better

**Table:**

	AGE	SEX	EDU	SR-IR	LF	DSB	VLT-DR
AGE	1	0.027	0.005	−0.408	0.11	−0.382	−0.315
SEX	0.027	1	−0.234	0.062	−0.026	−0.006	0.344
EDU	0.005	−0.234	1	0.045	0.279	0.074	0.072
SR-IR	−0.408	0.062	0.045	1	−0.114	0.16	0.4
LF	0.11	−0.026	0.279	−0.114	1	0.13	0.117
DSB	−0.382	−0.006	0.074	0.16	0.13	1	0.253
VLT-DR	−0.315	0.344	0.072	0.4	0.117	0.253	1

Fortin, A., & Caza, N. (2014). A validation study of memory and executive functions indexes in French-speaking healthy young and older adults. *Canadian Journal on Aging/La Revue canadienne du vieillissement*, *33*(1), 60–71. doi:10.1017/S0714980813000445

*N* = 98

Sex coding: female > male

Education coding: higher is better

**Table:**

	AGE	SEX	EDU	LF	DSF	DSB	COD	VLT-TR	VLT-DR
AGE	1	0.261	−0.097	0.196	−0.102	−0.025	−0.191	−0.442	−0.359
SEX	0.261	1	−0.046	0.196	0.037	−0.056	0.383	−0.007	−0.1
EDU	−0.097	−0.046	1	0.127	0.029	0.383	0.397	0.247	0.15
LF	0.196	0.196	0.127	1	0.11	0.441	0.18	0.083	0.092
DSF	−0.102	0.037	0.029	0.11	1	0.244	0.275	0.232	0.189
DSB	−0.025	−0.056	0.383	0.441	0.244	1	0.249	0.05	0.192
COD	−0.191	0.383	0.397	0.18	0.275	0.249	1	0.312	0.316
VLT-TR	−0.442	−0.007	0.247	0.083	0.232	0.05	0.312	1	0.817
VLT-DR	−0.359	−0.1	0.15	0.092	0.189	0.192	0.316	0.817	1

Gallagher, P., Gray, J. M., Watson, S., Young, A. H., & Ferrier, I. N. (2014). Neurocognitive functioning in bipolar depression: a component structure analysis. *Psychological Medicine*, *44*(5), 961–974. doi:10.1017/S0033291713001487

*N* = 47

Sex coding: female > male

Education coding: higher is better

**Table:**

	AGE	SEX	EDU	SF	VLT-TR
AGE	1	−0.08	−0.09	−0.21	−0.26
SEX	−0.08	1	−0.32	0.01	0.27
EDU	−0.09	−0.32	1	0.3	0.3
SF	−0.21	0.01	0.3	1	0.4
VLT-TR	−0.26	0.27	0.3	0.4	1

Horvat, P., Richards, M., Malyutina, S., Pajak, A., Kubinova, R., Tamosiunas, A., ... & Bobak, M. (2014). Life course socioeconomic position and mid-late life cognitive function in Eastern Europe. *Journals of Gerontology Series B: Psychological Sciences and Social Sciences*, *69*(3), 470–481. doi:10.1093/geronb/gbu014

Country: Czech Republic

*N* = 5490

Sex coding: female > male

Education coding: higher is better

**Table:**

	AGE	SEX	EDU	SF	VLT-TR
AGE	1	−0.02	−0.21	−0.29	−0.37
SEX	−0.02	1	−0.01	−0.01	0.28
EDU	−0.21	−0.01	1	0.4	0.43
SF	−0.29	−0.01	0.4	1	0.4
VLT-TR	−0.37	0.28	0.43	0.4	1

Horvat, P., Richards, M., Malyutina, S., Pajak, A., Kubinova, R., Tamosiunas, A., ... & Bobak, M. (2014). Life course socioeconomic position and mid-late life cognitive function in Eastern Europe. *Journals of Gerontology Series B: Psychological Sciences and Social Sciences*, *69*(3), 470–481. doi:10.1093/geronb/gbu014

Country: Lithuania

*N* = 6762

Sex coding: female > male

Education coding: higher is better

**Table:**

	AGE	SEX	EDU	SF	VLT-TR
AGE	1	−0.05	−0.11	−0.29	−0.36
SEX	−0.05	1	−0.09	0	0.16
EDU	−0.11	−0.09	1	0.38	0.36
SF	−0.29	0	0.38	1	0.55
VLT-TR	−0.36	0.16	0.36	0.55	1

Horvat, P., Richards, M., Malyutina, S., Pajak, A., Kubinova, R., Tamosiunas, A., ... & Bobak, M. (2014). Life course socioeconomic position and mid-late life cognitive function in Eastern Europe. *Journals of Gerontology Series B: Psychological Sciences and Social Sciences*, *69*(3), 470–481. doi:10.1093/geronb/gbu014

Country: Poland

*N* = 10,317

Sex coding: female > male

Education coding: higher is better

**Table:**

	AGE	SEX	EDU	SF	VLT-TR
AGE	1	−0.02	−0.17	−0.38	−0.42
SEX	−0.02	1	−0.04	−0.03	0.17
EDU	−0.17	−0.04	1	0.28	0.29
SF	−0.38	−0.03	0.28	1	0.47
VLT-TR	−0.42	0.17	0.29	0.47	1

Horvat, P., Richards, M., Malyutina, S., Pajak, A., Kubinova, R., Tamosiunas, A., ... & Bobak, M. (2014). Life course socioeconomic position and mid-late life cognitive function in Eastern Europe. *Journals of Gerontology Series B: Psychological Sciences and Social Sciences*, *69*(3), 470–481. doi:10.1093/geronb/gbu014

Country: Russia

*N* = 8277

Sex coding: female > male

Education coding: higher is better

**Table:**

	AGE	SEX	EDU	TMTA	TMTB	DSB
AGE	1	−0.16	0.14	0.34	0.34	−0.19
SEX	−0.16	1	−0.21	−0.07	0.01	−0.07
EDU	0.14	−0.21	1	0.05	−0.11	0.25
TMTA	0.34	−0.07	0.05	1	0.57	−0.23
TMTB	0.34	0.01	−0.11	0.57	1	−0.39
DSB	−0.19	−0.07	0.25	−0.23	−0.39	1

Hedden, T., & Yoon, C. (2006). Individual differences in executive processing predict susceptibility to interference in verbal working memory. *Neuropsychology*, *20*(5), 511–528. doi:10.1037/0894-4105.20.5.511.supp

*N* = 121

Sex coding: female > male

Education coding: higher is better

**Table:**

	AGE	SEX	EDU	TMTA	TMTB	LF	SF	DSB	COD
AGE	1	−0.04	−0.05	0.12	0.22	0.01	−0.19	−0.03	−0.22
SEX	−0.04	1	−0.09	0.07	0.14	0.03	0.06	−0.15	0.06
EDU	−0.05	−0.09	1	−0.22	−0.38	0.35	0.36	0.3	0.3
TMTA	0.12	0.07	−0.22	1	0.46	−0.13	−0.3	−0.12	−0.53
TMTB	0.22	0.14	−0.38	0.46	1	−0.35	−0.4	−0.28	−0.53
LF	0.01	0.03	0.35	−0.13	−0.35	1	0.56	0.35	0.38
SF	−0.19	0.06	0.36	−0.3	−0.4	0.56	1	0.33	0.48
DSB	−0.03	−0.15	0.3	−0.12	−0.28	0.35	0.33	1	0.28
COD	−0.22	0.06	0.3	−0.53	−0.53	0.38	0.48	0.28	1

Hedden, T., Mormino, E. C., Amariglio, R. E., Younger, A. P., Schultz, A. P., Becker, J. A., ... & Rentz, D. M. (2012). Cognitive profile of amyloid burden and white matter hyperintensities in cognitively normal older adults. *Journal of Neuroscience*, *32*(46), 16,233–16,242. doi:10.1523/JNEUROSCI.2462-12.2012

*N* = 168

Sex coding: female > male

Education coding: higher is better

**Table:**

	AGE	SEX	EDU	SR-IR	SR-DR
AGE	1	0.039	−0.552	−0.315	−0.279
SEX	0.039	1	0.068	−0.143	−0.13
EDU	−0.552	0.068	1	0.578	0.489
SR-IR	−0.315	−0.143	0.578	1	0.896
SR-DR	−0.279	−0.13	0.489	0.896	1

Hueng, T. T., Lee, I. H., Guog, Y. J., Chen, K. C., Chen, S. S., Chuang, S. P., ... & Yang, Y. K. (2011). Is a patient-administered depression rating scale valid for detecting cognitive deficits in patients with major depressive disorder? *Psychiatry and Clinical Neurosciences*, *65*(1), 70–76. doi:10.1111/j.1440-1819.2010.02166.x

*N* = 40

Sex coding: male > female

Education coding: higher is better

**Table:**

	AGE	SEX	EDU	TMTA	TMTB	LF	SF
AGE	1	0.015	0.3	0.022	0.077	0.151	0.126
SEX	0.015	1	0.264	0.042	0.009	0.218	0.091
EDU	0.3	0.264	1	−0.28	−0.314	0.415	0.409
TMTA	0.022	0.042	−0.28	1	0.582	−0.239	−0.19
TMTB	0.077	0.009	−0.314	0.582	1	−0.187	−0.207
LF	0.151	0.218	0.415	−0.239	−0.187	1	0.491
SF	0.126	0.091	0.409	−0.19	−0.207	0.491	1

Karagiannopoulou, L., Karamaouna, P., Zouraraki, C., Roussos, P., Bitsios, P., & Giakoumaki, S. G. (2016). Cognitive profiles of schizotypal dimensions in a community cohort: Common properties of differential manifestations. *Journal of Clinical and Experimental Neuropsychology*, *38*(9), 1050–1063. doi:10.1080/13803395.2016.1188890

*N* = 200

Sex coding: female > male

Education coding: higher is better

**Table:**

	AGE	SEX	EDU	TMTA	TMTB	LF	SF	DSF	DSB
AGE	1	−0.11	−0.1	0.25	0.24	−0.08	−0.16	−0.09	−0.06
SEX	−0.11	1	−0.04	0.01	0.02	0.12	0.04	−0.06	−0.01
EDU	−0.1	−0.04	1	−0.15	−0.28	0.29	0.27	0.18	0.2
TMTA	0.25	0.01	−0.15	1	0.49	−0.18	−0.23	−0.12	−0.15
TMTB	0.24	0.02	−0.28	0.49	1	−0.32	−0.32	−0.25	−0.31
LF	−0.08	0.12	0.29	−0.18	−0.32	1	0.5	0.26	0.27
SF	−0.16	0.04	0.27	−0.23	−0.32	0.5	1	0.22	0.24
DSF	−0.09	−0.06	0.18	−0.12	−0.25	0.26	0.22	1	0.46
DSB	−0.06	−0.01	0.2	−0.15	−0.31	0.27	0.24	0.46	1

Kesse-Guyot, E., Andreeva, V. A., Lassale, C., Hercberg, S., & Galan, P. (2014). Clustering of midlife lifestyle behaviors and subsequent cognitive function: a longitudinal study. *American Journal of Public Health*, *104*(11), 170–177. doi:10.2105/AJPH.2014.302121

*N* = 2470

Sex coding: female > male

Education coding: higher is better

**Table:**

	AGE	SEX	EDU	SF	VLT-TR	VLT-DR
AGE	1	−0.01	−0.255	−0.256	−0.337	−0.317
SEX	−0.01	1	−0.409	−0.216	0.182	0.25
EDU	−0.255	−0.409	1	0.37	0.2	0.049
SF	−0.256	−0.216	0.37	1	0.263	0.258
VLT-TR	−0.337	0.182	0.2	0.263	1	0.642
VLT-DR	−0.317	0.25	0.049	0.258	0.642	1

Kim, J., Jeong, J. H., Han, S. H., Ryu, H. J., Lee, J. Y., Ryu, S. H., ... & Choi, S. H. (2013). Relia;bility and validity of the short form of the literacy-independent cognitive assessment in the elderly. *Journal of Clinical Neurology*, *9*(2), 111–117. doi:10.3988/jcn.2013.9.2.111

*N* = 639

Sex coding: female > male

Education coding: higher is better

**Table:**

	AGE	SEX	EDU	SF	BNT	VLT-TR	VLT-DR
AGE	1	0.028	−0.194	−0.189	−0.208	−0.265	−0.24
SEX	0.028	1	0.026	−0.01	−0.23	0.219	0.168
EDU	−0.194	0.026	1	0.267	0.347	0.358	0.291
SF	−0.189	−0.01	0.267	1	0.375	0.401	0.343
BNT	−0.208	−0.23	0.347	0.375	1	0.253	0.238
VLT-TR	−0.265	0.219	0.358	0.401	0.253	1	0.746
VLT-DR	−0.24	0.168	0.291	0.343	0.238	0.746	1

Komulainen, P., Pedersen, M., Hänninen, T., Bruunsgaard, H., Lakka, T. A., Kivipelto, M., ... & Rauramaa, R. (2008). BDNF is a novel marker of cognitive function in ageing women: the DR’s EXTRA Study. *Neurobiology of Learning and Memory*, *90*(4), 596–603. doi:10.1016/j.nlm.2008.07.014

*N* = 1388

Sex coding: female > male

Education coding: higher is better

**Table:**

	AGE	SEX	EDU	SR-IR	SR-DR	SF	DSF	DSB	COD	BNT	VLT-TR	VLT-DR
AGE	1	0.07	0.092	−0.393	−0.304	−0.346	−0.141	−0.044	−0.095	−0.037	−0.352	−0.27
SEX	0.07	1	−0.019	−0.164	−0.203	−0.047	0.097	−0.097	−0.106	0.06	−0.106	−0.088
EDU	0.092	−0.019	1	0.058	0.208	0.147	0.036	0.243	0.371	0.459	0.259	0.273
SR-IR	−0.393	−0.164	0.058	1	0.89	0.467	0.055	0.41	0.373	0.445	0.591	0.557
SR-DR	−0.304	−0.203	0.208	0.89	1	0.403	0.063	0.42	0.464	0.512	0.62	0.573
SF	−0.346	−0.047	0.147	0.467	0.403	1	0.201	0.411	0.428	0.444	0.557	0.502
DSF	−0.141	0.097	0.036	0.055	0.063	0.201	1	0.303	0.103	0.229	0.253	0.072
DSB	−0.044	−0.097	0.243	0.41	0.42	0.411	0.303	1	0.467	0.407	0.422	0.27
COD	−0.095	−0.106	0.371	0.373	0.464	0.428	0.103	0.467	1	0.579	0.424	0.386
BNT	−0.037	0.06	0.459	0.445	0.512	0.444	0.229	0.407	0.579	1	0.478	0.516
VLT-TR	−0.352	−0.106	0.259	0.591	0.62	0.557	0.253	0.422	0.424	0.478	1	0.656
VLT-DR	−0.27	−0.088	0.273	0.557	0.573	0.502	0.072	0.27	0.386	0.516	0.656	1

Krueger, K. R., Wilson, R. S., Bennett, D. A., & Aggarwal, N. T. (2009). A battery of tests for assessing cognitive function in older Latino persons. *Alzheimer Disease and Associated Disorders*, *23*(4), 384. doi:10.1097/WAD.0b013e31819e0bfc

*N* = 66

Sex coding: male > female

Education coding: higher is better

**Table:**

	AGE	SEX	EDU	LF	SF
AGE	1	0.132	−0.347	−0.218	−0.452
SEX	0.132	1	−0.123	0.012	−0.016
EDU	−0.347	−0.123	1	0.352	0.355
LF	−0.218	0.012	0.352	1	0.498
SF	−0.452	−0.016	0.355	0.498	1

Laukka, E. J., Lövdén, M., Herlitz, A., Karlsson, S., Ferencz, B., Pantzar, A., ... & Bäckman, L. (2013). Genetic effects on old-age cognitive functioning: a population-based study. *Psychology and Aging*, *28*(1), 262. doi:10.1037/a0030829

*N* = 2694

Sex coding: female > male

Education coding: higher is better

**Table:**

	AGE	SEX	EDU	TMTA	TMTB	LF	SF	COD	BNT
AGE	1	0.016	−0.213	0.309	0.367	−0.128	−0.282	−0.427	−0.169
SEX	0.016	1	−0.117	0.148	0.128	−0.162	−0.27	−0.06	−0.036
EDU	−0.213	−0.117	1	−0.177	−0.293	0.288	0.178	0.206	0.101
TMTA	0.309	0.148	−0.177	1	0.661	−0.385	−0.371	−0.566	−0.197
TMTB	0.367	0.128	−0.293	0.661	1	−0.399	−0.362	−0.599	−0.199
LF	−0.128	−0.162	0.288	−0.385	−0.399	1	0.496	0.478	0.194
SF	−0.282	−0.27	0.178	−0.371	−0.362	0.496	1	0.514	0.316
COD	−0.427	−0.06	0.206	−0.566	−0.599	0.478	0.514	1	0.233
BNT	−0.169	−0.036	0.101	−0.197	−0.199	0.194	0.316	0.233	1

Lehrner, J., Moser, D., Klug, S., Gleiss, A., Auff, E., Pirker, W., & Pusswald, G. (2014). Subjective memory complaints, depressive symptoms and cognition in Parkinson’s disease patients. *European Journal of Neurology*, *21*(10), 1276–1285. doi:10.1111/ene.12470

N = 247

Sex coding: female > male

Education coding: higher is better

**Table:**

	AGE	SEX	EDU	TMTB	LF	SF	COD
AGE	1	0.006	−0.152	0.54	−0.15	−0.45	−0.447
SEX	0.006	1	−0.159	−0.068	0.278	0.158	0.141
EDU	−0.152	−0.159	1	−0.198	0.029	0.193	0.179
TMTB	0.54	−0.068	−0.198	1	−0.366	−0.474	−0.627
LF	−0.15	0.278	0.029	−0.366	1	0.618	0.324
SF	−0.45	0.158	0.193	−0.474	0.618	1	0.465
COD	−0.447	0.141	0.179	−0.627	0.324	0.465	1

Liebel, S. W., Jones, E. C., Oshri, A., Hallowell, E. S., Jerskey, B. A., Gunstad, J., & Sweet, L. H. (2017). Cognitive processing speed mediates the effects of cardiovascular disease on executive functioning. *Neuropsychology*, *31*(1), 44–51. doi:10.1037/neu0000324

*N* = 73

Sex coding: female > male

Education coding: higher is better

**Table:**

	AGE	SEX	EDU	TMTA	TMTB	LF	SF	DSF	DSB	COD
AGE	1	−0.035	0.132	0.393	0.415	−0.152	−0.235	−0.158	−0.179	−0.469
SEX	−0.035	1	0.134	0.083	0.086	−0.069	−0.069	−0.086	−0.135	−0.041
EDU	0.132	0.134	1	0.32	0.432	−0.312	−0.257	−0.303	−0.342	−0.522
TMTA	0.393	0.083	0.32	1	0.701	−0.304	−0.325	−0.286	−0.344	−0.654
TMTB	0.415	0.086	0.432	0.701	1	−0.393	−0.361	−0.379	−0.472	−0.708
LF	−0.152	−0.069	−0.312	−0.304	−0.393	1	0.466	0.316	0.368	0.422
SF	−0.235	−0.069	−0.257	−0.325	−0.361	0.466	1	0.238	0.302	0.401
DSF	−0.158	−0.086	−0.303	−0.286	−0.379	0.316	0.238	1	0.573	0.387
DSB	−0.179	−0.135	−0.342	−0.344	−0.472	0.368	0.302	0.573	1	0.458
COD	−0.469	−0.041	−0.522	−0.654	−0.708	0.422	0.401	0.387	0.458	1

Llinàs-Reglà, J., Vilalta-Franch, J., López-Pousa, S., Calvó-Perxas, L., Torrents Rodas, D., & Garre-Olmo, J. (2017). The trail making test: Association with other neuropsychological measures and normative values for adults aged 55 years and older From a Spanish-speaking population-based sample. *Assessment*, *24*(2), 183–196. doi:10.1177/1073191115602552

*N* = 1923

Sex coding: female > male

Education coding: **lower is better**

**Table:**

	AGE	SEX	EDU	TMTA	SF	COD	VLT-TR
AGE	1	−0.007	0.262	0.329	0.089	−0.473	−0.289
SEX	−0.007	1	−0.176	0.002	−0.104	−0.236	−0.166
EDU	0.262	−0.176	1	0.062	0.139	0.037	0.063
TMTA	0.329	0.002	0.062	1	−0.176	−0.503	−0.225
SF	0.089	−0.104	0.139	−0.176	1	0.191	0.25
COD	−0.473	−0.236	0.037	−0.503	0.191	1	0.449
VLT-TR	−0.289	−0.166	0.063	−0.225	0.25	0.449	1

Mohn, C., Lystad, J. U., Ueland, T., Falkum, E., & Rund, B. R. (2017). Factor analyzing the Norwegian MATRICS consensus cognitive battery. *Psychiatry and Clinical Neurosciences*, *71*(5), 336–345. doi:10.1111/pcn.12513

*N* = 300

Sex coding: male > female

Education coding: higher is better

**Table:**

	AGE	SEX	EDU	VLT-TR	VLT-DR
AGE	1	0.182	−0.04	−0.04	0.17
SEX	0.182	1	0	−0.049	−0.024
EDU	−0.04	0	1	−0.011	−0.237
VLT-TR	−0.04	−0.049	−0.011	1	0.719
VLT-DR	0.17	−0.024	−0.237	0.719	1

Morrens, M., Hulstijn, W., Matton, C., Madani, Y., Van Bouwel, L., Peuskens, J., & Sabbe, B. G. C. (2008). Delineating psychomotor slowing from reduced processing speed in schizophrenia. *Cognitive Neuropsychiatry*, *13*(6), 457–471. doi:10.1080/13546800802439312

N = 26

Sex coding: female > male

Education coding: higher is better

**Table:**

	AGE	SEX	EDU	TMTA	SR-IR	SR-DR	DSB	COD
AGE	1	−0.172	−0.243	0.625	−0.04	−0.053	−0.441	−0.589
SEX	−0.172	1	0.168	0.103	0.334	0.33	0.134	0.254
EDU	−0.243	0.168	1	−0.12	0.354	0.353	0.235	0.326
TMTA	0.625	0.103	−0.12	1	−0.05	−0.044	−0.502	−0.46
SR-IR	−0.04	0.334	0.354	−0.05	1	0.962	0.19	0.275
SR-DR	−0.053	0.33	0.353	−0.044	0.962	1	0.156	0.305
DSB	−0.441	0.134	0.235	−0.502	0.19	0.156	1	0.294
COD	−0.589	0.254	0.326	−0.46	0.275	0.305	0.294	1

Ojeda, N., Pena, J., Schretlen, D. J., Sanchez, P., Aretouli, E., Elizagarate, E., ... & Gutierrez, M. (2012). Hierarchical structure of the cognitive processes in schizophrenia: the fundamental role of processing speed. *Schizophrenia Research*, *135*(1), 72–78. doi:10.1016/j.schres.2011.12.004

*N* = 53

Sex coding: female > male

Education coding: higher is better

**Table:**

	AGE	SEX	EDU	LF	SF	DSF	DSB	VLT-TR	VLT-DR
AGE	1	−0.141	−0.163	0.016	−0.085	−0.111	−0.179	−0.153	−0.018
SEX	−0.141	1	0.274	0.091	0.305	0.212	0.098	0.176	0.254
EDU	−0.163	0.274	1	0.411	0.564	0.211	0.401	0.447	0.309
LF	0.016	0.091	0.411	1	0.649	0.332	0.321	0.406	0.409
SF	−0.085	0.305	0.564	0.649	1	0.247	0.35	0.537	0.596
DSF	−0.111	0.212	0.211	0.332	0.247	1	0.246	0.34	0.256
DSB	−0.179	0.098	0.401	0.321	0.35	0.246	1	0.101	0.157
VLT-TR	−0.153	0.176	0.447	0.406	0.537	0.34	0.101	1	0.689
VLT-DR	−0.018	0.254	0.309	0.409	0.596	0.256	0.157	0.689	1

De Paula, J. J., Bertola, L., Avila, R. T., Moreira, L., Coutinho, G., de Moraes, E. N., ... & Malloy-Diniz, L. F. (2013). Clinical applicability and cutoff values for an unstructured neuropsychological assessment protocol for older adults with low formal education. *PLoS One*, *8*(9), 1–9. 10.1371/journal.pone.0073167

*N* = 96

Sex coding: female > male

Education coding: higher is better

**Table:**

	AGE	SEX	EDU	TMTA	TMTB	SR-DR	LF	SF	BNT	VLT-TR	VLT-DR
AGE	1	0.037	−0.088	0.279	0.347	−0.137	−0.13	−0.255	−0.254	−0.246	−0.208
SEX	0.037	1	−0.157	0.066	0.06	0.13	−0.043	0.018	−0.079	0.258	0.229
EDU	−0.088	−0.157	1	−0.118	−0.208	0.212	0.39	0.262	0.193	0.148	−0.023
TMTA	0.279	0.066	−0.118	1	0.511	−0.039	−0.124	−0.193	−0.15	−0.117	−0.137
TMTB	0.347	0.06	−0.208	0.511	1	−0.129	−0.326	−0.274	−0.195	−0.231	−0.122
SR-DR	−0.137	0.13	0.212	−0.039	−0.129	1	0.1	0.228	0.167	0.388	0.314
LF	−0.13	−0.043	0.39	−0.124	−0.326	0.1	1	0.318	0.264	0.192	0.095
SF	−0.255	0.018	0.262	−0.193	−0.274	0.228	0.318	1	0.299	0.259	0.126
BNT	−0.254	−0.079	0.193	−0.15	−0.195	0.167	0.264	0.299	1	0.197	0.151
VLT-TR	−0.246	0.258	0.148	−0.117	−0.231	0.388	0.192	0.259	0.197	1	0.756
VLT-DR	−0.208	0.229	−0.023	−0.137	−0.122	0.314	0.095	0.126	0.151	0.756	1

Reppermund, S., Sachdev, P. S., Crawford, J., Kochan, N. A., Slavin, M. J., Kang, K., ... & Brodaty, H. (2011). The relationship of neuropsychological function to instrumental activities of daily living in mild cognitive impairment. *International Journal of Geriatric Psychiatry*, *26*(8), 843–852. doi:10.1002/gps.2612

*N* = 469

Sex coding: female > male

Education coding: higher is better

**Table:**

	AGE	SEX	EDU	LF	SF
AGE	1	0.138	−0.886	−0.37	−0.545
SEX	0.138	1	0.011	0.089	0.01
EDU	−0.886	0.011	1	0.291	0.436
LF	−0.37	0.089	0.291	1	0.669
SF	−0.545	0.01	0.436	0.669	1

Ricarte, J. J., Ros, L., Latorre, J. M., Muñoz, M. D., Aguilar, M. J., & Hernandez, J. V. (2016). Role of anxiety and brooding in specificity of autobiographical recall. *Scandinavian Journal of Psychology*, *57*(6), 495–500. doi:10.1111/sjop.12323

*N* = 210

Sex coding: male > female

Education coding: higher is better

**Table:**

Royall, D. R., Bishnoi, R. J., & Palmer, R. F. (2015). Serum IGF-BP2 strongly moderates age’s effect on cognition: a MIMIC analysis. *Neurobiology of Aging*, *36*(7), 2232–2240. doi:10.1016/j.neurobiolaging.2015.04.003	
*N* = 875	
Sex coding: female > male	
Education coding: higher is better

**Table:**

Schmidt, C. S., Schumacher, L. V., Römer, P., Leonhart, R., Beume, L., Martin, M., ... & Kaller, C. P. (2017). Are semantic and phonological fluency based on the same or distinct sets of cognitive processes? Insights from factor analyses in healthy adults and stroke patients. *Neuropsychologia*, *99*, 148–155. doi:10.1016/j.neuropsychologia.2017.02.019					
Sex coding: female > male					
Education coding: higher is better					
	AGE	SEX	EDU	LF	SF
AGE	1	−0.001	0.855	0.003	0.25
SEX	−0.001	1	−0.041	0.194	0.133
EDU	0.855	−0.041	1	−0.032	0.189
LF	0.003	0.194	−0.032	1	0.521
SF	0.25	0.133	0.189	0.521	1

**Table:**

Siedlecki, K. L., Manly, J. J., Brickman, A. M., Schupf, N., Tang, M. X., & Stern, Y. (2010). Do neuropsychological tests have the same meaning in Spanish speakers as they do in English speakers?. *Neuropsychology*, *24(3)*, 402–411. doi:10.1037/a0017515	
*N* = 2113	
Sex coding: female > male	
Education coding: higher is better

**Table:**

	AGE	EDU	TMTA	TMTB	SR-IR	SR-DR	LF	SF	DSF	BNT
AGE	1	−0.189	0.357	0.417	−0.318	−0.289	−0.188	−0.325	−0.162	−0.3
EDU	−0.189	1	−0.107	−0.187	0.186	0.168	0.2	0.168	0.12	0.232
TMTA	0.357	−0.107	1	0.546	−0.098	−0.07	−0.192	−0.256	−0.154	−0.247
TMTB	0.417	−0.187	0.546	1	−0.3	−0.268	−0.28	−0.364	−0.242	−0.343
SR-IR	−0.318	0.186	−0.098	−0.3	1	0.872	0.246	0.364	0.225	0.391
SR-DR	−0.289	0.168	−0.07	−0.268	0.872	1	0.243	0.357	0.203	0.376
LF	−0.188	0.2	−0.192	−0.28	0.246	0.243	1	0.487	0.239	0.356
SF	−0.325	0.168	−0.256	−0.364	0.364	0.357	0.487	1	0.217	0.452
DSF	−0.162	0.12	−0.154	−0.242	0.225	0.203	0.239	0.217	1	0.222
BNT	−0.3	0.232	−0.247	−0.343	0.391	0.376	0.356	0.452	0.222	1

Snitz, B. E., Yu, L., Crane, P. K., Chang, C. C. H., Hughes, T. F., & Ganguli, M. (2012). Subjective cognitive complaints of older adults at the population level: an item response theory analysis. *Alzheimer Disease and Associated Disorders*, *26*(4), 344–351. doi:10.1097/WAD.0b013e3182420bdf

*N* = 1356

Sex coding: Sex not included

Education coding: higher is better

**Table:**

	AGE	SEX	EDU	SF	BNT	VLT-TR	VLT-DR
AGE	1	−0.042	−0.428	−0.425	−0.509	−0.546	−0.533
SEX	−0.042	1	0.037	−0.024	−0.072	0.211	0.169
EDU	−0.428	0.037	1	0.518	0.584	0.567	0.496
SF	−0.425	−0.024	0.518	1	0.546	0.559	0.524
BNT	−0.509	−0.072	0.584	0.546	1	0.6	0.53
VLT-TR	−0.546	0.211	0.567	0.559	0.6	1	0.801
VLT-DR	−0.533	0.169	0.496	0.524	0.53	0.801	1

Tractenberg, R. E., Fillenbaum, G., Aisen, P. S., Liebke, D. E., Yumoto, F., & Kuchibhatla, M. N. (2010). What the CERAD battery can tell us about executive function as a higher-order cognitive faculty. *Current Gerontology and Geriatrics Research*, *510,614*, 1–10. doi:10.1155/2010/510614

*N* = 918

Sex coding: female > male

Education coding: higher is better

**Table:**

	AGE	SEX	EDU	TMTA	TMTB	SR-IR	LF	SF	DSF	DSB	COD	BNT
AGE	1	−0.061	0.021	0.342	0.355	−0.157	0.044	−0.245	0.056	−0.002	−0.284	−0.007
SEX	−0.061	1	−0.159	0.008	0.019	0.015	−0.011	−0.083	−0.03	0.1	0.182	0.049
EDU	0.021	−0.159	1	−0.134	−0.205	0.247	0.284	0.199	0.104	0.063	0.119	0.102
TMTA	0.342	0.008	−0.134	1	0.673	−0.238	−0.277	−0.354	−0.045	−0.14	−0.582	−0.071
TMTB	0.355	0.019	−0.205	0.673	1	−0.251	−0.257	−0.251	−0.162	−0.271	−0.508	−0.03
SR-IR	−0.157	0.015	0.247	−0.238	−0.251	1	0.186	0.246	0.074	0.195	0.23	0.097
LF	0.044	−0.011	0.284	−0.277	−0.257	0.186	1	0.394	0.234	0.312	0.355	0.08
SF	−0.245	−0.083	0.199	−0.354	−0.251	0.246	0.394	1	0.19	0.217	0.344	0.213
DSF	0.056	−0.03	0.104	−0.045	−0.162	0.074	0.234	0.19	1	0.485	0.053	0.105
DSB	−0.002	0.1	0.063	−0.14	−0.271	0.195	0.312	0.217	0.485	1	0.164	0.129
COD	−0.284	0.182	0.119	−0.582	−0.508	0.23	0.355	0.344	0.053	0.164	1	0.023
BNT	−0.007	0.049	0.102	−0.071	−0.03	0.097	0.08	0.213	0.105	0.129	0.023	1

Tse, C. S., Balota, D. A., Yap, M. J., Duchek, J. M., & McCabe, D. P. (2010). Effects of healthy aging and early stage dementia of the Alzheimer’s type on components of response time distributions in three attention tasks. *Neuropsychology*, *24*(3), 300–315. doi:10.1037/a0018274

*N* = 246

Sex coding: female > male

Education coding: higher is better

**Table:**

	AGE	SEX	EDU	LF	SF	COD	VLT-TR
AGE	1	0.07	0.06	−0.02	−0.16	−0.28	−0.3
SEX	0.07	1	0.05	0.15	−0.06	0.07	0.25
EDU	0.06	0.05	1	0.51	0.33	0.51	0.3
LF	−0.02	0.15	0.51	1	0.48	0.58	0.42
SF	−0.16	−0.06	0.33	0.48	1	0.51	0.37
COD	−0.28	0.07	0.51	0.58	0.51	1	0.52
VLT-TR	−0.3	0.25	0.3	0.42	0.37	0.52	1

Tuokko, H. A., Chou, P. H. B., Bowden, S. C., Simard, M., Ska, B., & Crossley, M. (2009). Partial measurement equivalence of French and English versions of the Canadian Study of Health and Aging neuropsychological battery. *Journal of the International Neuropsychological Society*, *15*(3), 416–425. 10.1017/S1355617709090602

*N* = 786

Sex coding: female > male

Education coding: higher is better

**Table:**

	AGE	SEX	EDU	TMTA	TMTB	SR-IR	SR-DR	LF	SF	DSF	DSB	COD	BNT
AGE	1	0.031	−0.168	0.459	0.493	−0.302	−0.362	−0.178	−0.47	0.072	−0.118	−0.482	−0.309
SEX	0.031	1	−0.3	0.139	0.017	0.078	0.011	0.227	0.07	−0.137	−0.097	−0.027	0.072
EDU	−0.168	−0.3	1	−0.185	−0.137	0.17	0.259	0.099	0.126	0.012	0.155	0.309	0.037
TMTA	0.459	0.139	−0.185	1	0.546	−0.039	−0.183	−0.056	−0.344	0.029	0.046	−0.457	−0.258
TMTB	0.493	0.017	−0.137	0.546	1	−0.289	−0.403	−0.244	−0.276	−0.142	−0.299	−0.606	−0.387
SR-IR	−0.302	0.078	0.17	−0.039	−0.289	1	0.895	0.154	0.231	0.097	0.325	0.39	0.425
SR-DR	−0.362	0.011	0.259	−0.183	−0.403	0.895	1	0.159	0.273	0.122	0.355	0.537	0.5
LF	−0.178	0.227	0.099	−0.056	−0.244	0.154	0.159	1	0.365	0.253	0.313	0.407	0.067
SF	−0.47	0.07	0.126	−0.344	−0.276	0.231	0.273	0.365	1	−0.107	0.074	0.398	0.284
DSF	0.072	−0.137	0.012	0.029	−0.142	0.097	0.122	0.253	−0.107	1	0.498	0.222	0.091
DSB	−0.118	−0.097	0.155	0.046	−0.299	0.325	0.355	0.313	0.074	0.498	1	0.249	0.221
COD	−0.482	−0.027	0.309	−0.457	−0.606	0.39	0.537	0.407	0.398	0.222	0.249	1	0.288
BNT	−0.309	0.072	0.037	−0.258	−0.387	0.425	0.5	0.067	0.284	0.091	0.221	0.288	1

Valenzuela, M. J., & Sachdev, P. (2007). Assessment of complex mental activity across the lifespan: development of the Lifetime of Experiences Questionnaire (LEQ). *Psychological Medicine*, *37*(7), 1015–1025. 10.1017/S003329170600938X

N = 73

Sex coding: female > male

Education coding: higher is better

**Table:**

	AGE	SEX	EDU	TMTA	TMTB	LF	SF	BNT	VLT-TR	VLT-DR
AGE	1	0.335	0.514	0.266	0.247	0.08	−0.269	−0.059	−0.053	−0.186
SEX	0.335	1	0.139	0.124	0.105	−0.136	−0.319	0.078	−0.121	−0.164
EDU	0.514	0.139	1	0.046	0.009	0.31	−0.008	0.033	0.227	0.064
TMTA	0.266	0.124	0.046	1	0.581	−0.336	−0.387	−0.224	−0.269	−0.293
TMTB	0.247	0.105	0.009	0.581	1	−0.338	−0.443	−0.126	−0.4	−0.408
LF	0.08	−0.136	0.31	−0.336	−0.338	1	0.553	0.108	0.443	0.318
SF	−0.269	−0.319	−0.008	−0.387	−0.443	0.553	1	0.301	0.559	0.521
BNT	−0.059	0.078	0.033	−0.224	−0.126	0.108	0.301	1	0.293	0.252
VLT-TR	−0.053	−0.121	0.227	−0.269	−0.4	0.443	0.559	0.293	1	0.74
VLT-DR	−0.186	−0.164	0.064	−0.293	−0.408	0.318	0.521	0.252	0.74	1

Waldinger, R. J., Cohen, S., Schulz, M. S., & Crowell, J. A. (2015). Security of attachment to spouses in late life: Concurrent and prospective links with cognitive and emotional well-being. *Clinical Psychological Science*, *3*(4), 516–529. doi:10.1177/2167702614541261

*N* = 240

Sex coding: male > female

Education coding: higher is better

**Table:**

	AGE	SEX	EDU	SR-IR	SR-DR
AGE	1	−0.051	−0.072	−0.264	−0.296
SEX	−0.051	1	0.284	−0.138	−0.165
EDU	−0.072	0.284	1	0.153	0.081
SR-IR	−0.264	−0.138	0.153	1	0.847
SR-DR	−0.296	−0.165	0.081	0.847	1

Watts, A. S., Loskutova, N., Burns, J. M., & Johnson, D. K. (2013). Metabolic syndrome and cognitive decline in early Alzheimer’s disease and healthy older adults. *Journal of Alzheimer’s Disease*, *35*(2), 253–265. doi:10.3233/JAD-121168

*N* = 73

Sex coding: male > female

Education coding: higher is better

**Table:**

	AGE	SEX	EDU	SF	DSB
AGE	1	0.077	0.124	−0.19	−0.061
SEX	0.077	1	−0.009	0.007	0.132
EDU	0.124	−0.009	1	0.262	0.124
SF	−0.19	0.007	0.262	1	0.198
DSB	−0.061	0.132	0.124	0.198	1

Wettstein, M., Kuźma, E., Wahl, H. W., & Heyl, V. (2016). Cross-sectional and longitudinal relationship between neuroticism and cognitive ability in advanced old age: The moderating role of severe sensory impairment. *Aging & Mental Health*, *20*(9), 918–929. 10.1080/13607863.2015.1049119

*N* = 150

Sex coding: female > male

Education coding: higher is better

**Table:**

Williams, P. G., Suchy, Y., & Kraybill, M. L. (2010). Five-factor model personality traits and executive functioning among older adults. *Journal of Research in Personality*, *44*(4), 485–491. doi:10.1016/j.jrp.2010.06.002	
*N* = 62	
Sex coding: female > male	
Education coding: higher is better	
