# Supplementary material for: The Factor Structure of Cognitive Functioning in Cognitively Healthy Participants: a Meta-Analysis and Meta-Analysis of Individual Participant Data
Source: Neuropsychol Rev. 2020 Feb 1;30(1):51–96. doi: 10.1007/s11065-019-09423-6 (PMC7089912; doi:10.1007/s11065-019-09423-6)
Supplement: Supplementary file 1 — (DOCX 15 kb) [file 11065_2019_9423_MOESM1_ESM.docx]

Supplemental Materials : Table of Citations of Contributing Studies

| Adrover-Roig et al., 2012 |
| --- |
| Andrejeva et al., 2016 |
| Andreotti & Hawkins, 2015 |
| Albert et al., 2010 |
| Barnes et al., 2016 |
| Bennett & Stark, 2016 |
| Bezdicek et al., 2014 |
| Booth et al., 2015 |
| Bouazzaoui et al., 2013 |
| Bowden et al., 2004 |
| Bunce, Batterham, Christensen, & Mackinnon, 2014 |
| Burns, Nettelbeck, & McPherson, 2009 |
| Chan et al., 2009 |
| Chen et al., 2017 |
| Ciccarelli et al., 2012 |
| Darst et al., 2015 |
| DeYoung, Peterson, & Higgins, 2005 |
| Duff et al., 2006 |
| Eifler et al., 2014 |
| Ferreira et al., 2015 |
| Fernaeus, Östberg, Wahlund, & Hellström, 2014 |
| Fortin & Caza, 2014 |
| Gallagher, Gray, Watson, Young, Ferrier, 2014 |
| Hedden & Yoon, 2006 |
| Hedden et al., 2014 |
| Horvat et al., 2014 |
| Hueng et al., 2011 |
| Kafadar, 2012 |
| Karagiannopoulou et al., 2016 |
| Kesse-Guyot, Andreeva, Lassale, Hercberg, & Galan, 2014 |
| Kim et al., 2013 |
| Komulainen et al., 2008 |
| Krueger, Wilson, Bennett, & Aggarwal, 2009 |
| Laukka et al., 2013 |
| Lehrner et al., 2014 |
| Liebel et al., 2017, Llinàs-Reglà et al., 2017 |
| Mohn, Lystad, Ueland, Falkum, & Rund, 2017 |
| Morrens et al., 2008 |
| Ojeda et al., 2012 |
| De Paula et al., 2013 |
| Reppermund et al., 2011 |
| Ricarte et al., 2016 |
| Royall, Bishnoi, & Palmer, 2015 |
| Schmidt et al., 2017, Siedlecki et al., 2010 |
| Snitz et al., 2015 |
| Sternäng, Lövdén, Kabir, Hamadani, & Wahlin, 2016 |
| Thibeau, McFall, Wiebe, Anstey, & Dixon, 2016 |
| Tractenberg et al., 2010 |
| Tse, Balota, Yap, Duchek, & McCabe, 2010 |
| Tuokko et al., 2009 |
| Valenzuela & Sachdev, 2007 |
| Waldinger, Cohen, Schulz, & Cromwell, 2015 |
| Watts, Loskutova, Burns, & Johnson, 2013 |
| Wettstein, Kuźma, Wahl, & Heyl, 2016 |
| Williams, Suchy, & Kraybill, 2010. |
