# Supplementary material for: The Factor Structure of Cognitive Functioning in Cognitively Healthy Participants: a Meta-Analysis and Meta-Analysis of Individual Participant Data
Source: Neuropsychol Rev. 2020 Feb 1;30(1):51–96. doi: 10.1007/s11065-019-09423-6 (PMC7089912; doi:10.1007/s11065-019-09423-6)
Supplement: Supplementary file 2 — (PDF 399 kb) [file 11065_2019_9423_MOESM2_ESM.pdf]

R, metaSEM and Mplus code

Stage 1 of Study 1, in R

```
# The psych and metaSEM packages were used for the analysis

# The full correlation matrix was reduced to the partial correlation matrix using the partial.r function from the psych package
partial.r( fullcormat, x = which(varnames %in% testnames), y = which(varnames %in% demonames))
# This is done for every study individually.
# The first argument is the full correlation matrix from a particular study.
# The second argument specifies which variables will be in the final partial correlation matrix (the test variables)
# The third argument specifies which variables will be partialled out, out of all other correlations (the demographic variables)
# All partial correlation matrices are then collected in a larger data object, called dat.

# dat is a list of 55 partial correlation matrices, of dimensions 12x12
# All data for which we received explicit permission to share are provided with the article.
# As an example of the structure of the dat object, consider the data from Andrejeva et al. (2016)
# dat[[2]]
#            SF       DSBackward VLTtotal  StoryImm  StoryDel LF      TMTb Coding DSForward      TMTa VLTrecall     BNT
# SF         1.000000         NA 0.322654 0.071495  0.118443 NA  0.266187     NA        NA  0.278264  0.212713 0.137812
# DSBackward       NA         NA       NA       NA        NA NA        NA     NA        NA        NA        NA       NA
# VLTtotal   0.322654         NA 1.000000 0.115081  0.088780 NA  0.254762     NA        NA  0.025220  0.519629 0.142994
# StoryImm   0.071495         NA 0.115081 1.000000  0.853113 NA  0.042010     NA        NA  0.054662  0.092655 0.261756
# StoryDel   0.118443         NA 0.088780 0.853113  1.000000 NA -0.054331     NA        NA  0.008251  0.080811 0.205960
# LF               NA         NA       NA       NA        NA NA        NA     NA        NA        NA        NA       NA
# TMTb       0.266187         NA 0.254762 0.042010 -0.054331 NA  1.000000     NA        NA  0.361753  0.073062 0.166820
# Coding           NA         NA       NA       NA        NA NA        NA     NA        NA        NA        NA       NA
# DSForward        NA         NA       NA       NA        NA NA        NA     NA        NA        NA        NA       NA
# TMTa       0.278264         NA 0.025220 0.054662  0.008251 NA  0.361753     NA        NA  1.000000 -0.154973 0.039681
# VLTrecall  0.212713         NA 0.519629 0.092655  0.080811 NA  0.073062     NA        NA -0.154973  1.000000 0.059691
# BNT        0.137812         NA 0.142994 0.261756  0.205960 NA  0.166820     NA        NA  0.039681  0.059691 1.000000
#
# N is a vector of integers of length 55
# As an example of the structure of the N object, consider the data from Andrejeva et al. (2016)
# > N[2]
# Data Andrejeva (nadeshda.andrejeva@gmail.com) Andrejeva2016 Neuro.csv
# 65

# To perform the first stage of the analysis, the tssem1 function from the metaSEM package is used.
# This function provides a weighted average of all the input partial correlation matrices, which
# will be further analyzed in following steps.
firststage <- tssem1(dat, N, method="REM", RE.type = "Zero")
```

Stage 2 of Study 1, for CHC model 2, in R

```
# Based on documentation

number_tests <- 12
test_names <- c("SF", "DSBackward", "VLTtotal", "StoryImm", "StoryDel", "LF",
"TMTb", "Coding", "DSForward", "TMTa", "VLTrecall", "BNT")

# Gc: BNT, StoryImm, StoryDel
# Gs: TMTa, TMTb, Coding,
# Glr: VLTtotal, VLTrecall, StoryImm, StoryDel
# Gsm: DSbackward, DSForward, TMTb
# FW: LF, SF

### Factor model specification
number_latent_factors <- 5
latent_factor_names <- c("Gc", "Gs", "Glr", "Gsm", "FW")

## Factor covariance among latent factors
PSI <- matrix(NA, ncol=number_latent_factors, nrow=number_latent_factors)
for( i in 1:number_latent_factors)
  for( j in i:number_latent_factors)
    PSI[i,j] <- PSI[j,i] <- paste0( "0.3*PSI", i, j)
diag( PSI) <- 1

## Residual covariance matrix
THETA <- Diag(paste0( "0.2*epsilon", 1:number_tests))

## Complete covariance matrix
SIGMA <- bdiagMat(list(THETA, PSI))
dimnames(SIGMA)[[1]] <- dimnames(SIGMA)[[2]] <- c(test_names,latent_factor_names)

## Factor loadings matrix (first initialized, then filled at the #appropriate coordinates)
LAMBDA <- matrix( 0, nrow = number_tests, ncol = number_latent_factors)
factor_loading_coordinates <-
  matrix( c(1,5,
            2,4,
            3,3,
            4,1,
            5,1,
            4,3,
            5,3,
```

```
            6,5,
            7,2,
            7,4,
            8,2,
            9,4,
            10,2,
            11,3,
            12,1), ncol = 2, byrow = T)
for( i in 1:nrow(factor_loading_coordinates)){
 LAMBDA[factor_loading_coordinates[i,1],factor_loading_coordinates[i,2]] <- paste0(".3*",
latent_factor_names[factor_loading_coordinates[i,2]], "_", test_names[factor_loading_coordinates[i,1]] )
}

## Inter-indicator loadings (irrelevant for our case, so stays at #initialization of 0)
inter_indicator_loadingsMAT <- matrix( 0, nrow = number_tests, ncol = number_tests)

## Covariate loadings (indicators influencing latent factors) #(irrelevant for our case, so stays at initialization of
0)
covariate_loadingsMAT <- matrix( 0, nrow = number_latent_factors, ncol = number_tests)

## Inter-factor loadings
#(irrelevant for our case, so stays at initialization of 0)
BETA <- matrix( 0, nrow = number_latent_factors, ncol = number_latent_factors)

## Complete loadings matrix
LOADINGS <- rbind( cbind( inter_indicator_loadingsMAT, LAMBDA),
                   cbind( covariate_loadingsMAT, BETA))
dimnames(LOADINGS)[[1]] <- dimnames(LOADINGS)[[2]] <- c(test_names,latent_factor_names)

## F matrix to select the observed variables
OBS <- create.Fmatrix(c(rep( 1, number_tests),rep( 0, number_latent_factors)), as.mxMatrix=FALSE)
### End model specification

secondstage <- tssem2( firststage, Amatrix=LOADINGS, Smatrix=SIGMA, Fmatrix=OBS, model.name="TSSEM2 BASIC",
intervals.type = "z")
summary(secondstage)
```

Mplus model specification for Study 2

```
TITLE: Full model MetaSEM Paper on ANDI
  DATA: FILE = "Mplusdatasetresiduals.dat";
  VARIABLE:
  NAMES =ID STUDY SEX EDU AGE AGESQ AVLTTR
```

```
 AVLTDR SRIMM SRDEL TMTA TMTB LF SF BNT
 CODING;
USEVARIABLES =
 AVLTTR AVLTDR SRIMM SRDEL TMTA TMTB LF
 SF BNT CODING;
MISSING = .;
ANALYSIS: COVERAGE = 0;

MODEL:
AVLTTR;
 AVLTDR;
 SRIMM;
 SRDEL;
 TMTA;
 TMTB;
 LF;
 SF;
 BNT;
 CODING;

Gc BY BNT* SRIMM SRDEL;
Gs BY TMTA* TMTB CODING;
Glr BY AVLTTR* AVLTDR SRIMM SRDEL;
FW BY LF* SF;

Gc@1;
Gs@1;
Glr@1;
FW@1
```
